# Enhancing English reading motivation and performance via the ARCS model: an empirical study using the ARCS motivation scale

**DOI:** 10.3389/fpsyg.2025.1499957

**Published:** 2025-10-28

**Authors:** Zhonger Wang, Jin Lu, Shenchen Yu

**Affiliations:** School of Foreign Languages, Shanghai Lixin University of Accounting and Finance, Shanghai, China

**Keywords:** ARCS, reading motivation, reading performance, Chinese university students, CET-4, English teaching approach

## Abstract

The ARCS motivation model comprises four factors: Attention, Relevance, Confidence, and Satisfaction. This study aims to investigate the effectiveness of the teaching approach based on this model in English reading for Chinese university students. Two classes of 80 sophomores participated in the experiment, with 40 in the experimental group and 40 in the control group. The experimental group received ARCS-based instruction, while the control group followed the traditional grammar-translation method over a four-month teaching session. A survey, which included the four factors derived from exploratory and confirmatory factor analysis, was conducted among 611 university students before and after the experiment to explore the English reading motivation of the two classes of students in four dimensions. Also, the research team administered two English reading tests of the same difficulty level to assess students’ reading ability before and after the experiment. The results of the data analysis showed that using an independent-sample *t-*test, there was a statistically significant increase in the total reading motivation level and a rise in the four dimensions for the students in the experimental group who received instruction based on the ARCS model. Additionally, when the researchers retested the total English reading scores of the experimental group using independent-samples *t* tests again, the total English reading scores of the experimental group increased significantly. Furthermore, all four factors were significantly correlated with English reading achievement, with Confidence and Satisfaction playing important roles in predicting their reading proficiency. The study concludes by outlining measures to improve the ARCS-based instructional approach.

## 1 Introduction

Reading ability reflects an individual’s ability to comprehend and use a language effectively. In China’s College English Test Band 4 (CET-4), the reading test accounts for 35% of the total score, which indicates that reading ability is of vital importance (Guo and Chen, 2007).

A key factor in foreign language (FL) learning is motivation. Muñoz-Restrepo et al. (2020) argued that motivation played a significant role in the effectiveness of FL teaching by influencing learners’ approaches and engagement. Similarly, Fandiño et al. (2019) claimed that motivation was a decisive element in the FL learning process. Meanwhile, reading motivation plays a key role in English reading among Chinese students. Al-Hoorie (2018) found a significant positive correlation between overall reading motivation and reading performance (2018). Likewise, Taguchi et al. (2009) argued that students would make less effort in reading due to their lack of motivation.

However, English reading instruction in Chinese universities remains primarily characterized by traditional grammar-translation methods, rigid instructional design, and passive student learning (Du, 2021). Since such a model suppresses learners’ motivation and hinders the development of their reading skills, it is advisable to develop new ways to stimulate university students’ reading motivation and enhance their reading performance in English.

In the 1980s, Keller developed the ARCS motivation model, which consists of four components: Attention, Relevance, Confidence, and Satisfaction (Keller, 1987a). Its wide application is in both motivation enhancement and instructional design. For instance, Han et al. (2024) maintained that the ARCS model was effective in enhancing the motivation of primary school students and improving their knowledge of China’s intangible cultural heritage.

Despite some empirical studies in Chinese elementary schools, there is still a lack of empirical research on the application of the ARCS model in English reading instruction at Chinese universities. Hence, this study aims to explore the impact of the English reading teaching approach based on the ARCS model on English reading motivation and performance among Chinese university students.

Due to the uniqueness of China’s English education environment, the ARCS model needs to be adjusted accordingly to accommodate the characteristics of students in this context. For example, in addition to internal interest, the evaluation system set by examination institutions to measure students’ academic performance also influences their English reading motivation, especially in standardized tests with significant influence such as CET and postgraduate entrance English exams, where test-takers’ scores are closely related to their opportunities for further education or employment (Bai, 2020; Chen et al., 2005). Therefore, when developing evaluation tools, it is essential to consider these cultural traits. To address this need, this study developed and validated a questionnaire on English reading motivation for Chinese college students. Based on the ARCS model as the theoretical foundation, this study designs a questionnaire to reflect the actual situation of Chinese college English learners, ensuring that the questionnaire items meet their characteristics and needs. This approach enables the more accurate identification of their specific characteristics in English reading motivation.

## 2 Theoretical framework

This section provides the theoretical foundation for the study, clarifying its basis. The research begins by introducing the ARCS motivation model, which serves as a conceptual framework to guide the design of instructional interventions and the development of a reading motivation scale. Subsequently, based on existing literature and the characteristics of Chinese university students, this study expounds on the concepts of reading motivation and reading ability.

### 2.1 ARCS motivation model

To better understand the key components of learning motivation and to develop systematic methods for addressing related issues, Keller developed the ARCS motivation model (Visser and Keller, 1990). Keller (1987b) considered the four elements in the instructional design. In addition, Keller’s theory elaborates on approaches to stimulating motivation, relevant teaching strategies, and application procedures.

#### 2.1.1 Elements of the ARCS motivation model

Attention, as the first element, refers to the attraction and maintenance of learners’ attention as well as the arousal and maintenance of their interest and curiosity. It is the prerequisite for learning, as practical teaching activities depend on the activation of students’ interest and curiosity in the learning task (Li and Keller, 2018).

Relevance refers to a close correlation between learning and the goals, needs, and interests of the students. Teachers can enhance students’ motivation by establishing such connections when presenting learning materials. Students, in turn, are more likely to be motivated if they perceive the tasks as relevant to their personal goals or contributing to their self-worth (Reynolds et al., 2017).

The next component is Confidence, which refers to learners’ belief in their abilities and future success, thereby motivating them to engage in learning (Li and Keller, 2018). Self-confident individuals are typically equipped with higher self-efficacy and achievement motivation, believing they can achieve their goals (Bandura, 1982).

The final component, Satisfaction, refers to the emotional experience learners derive from the learning process and the sense of fulfillment—both intrinsic and extrinsic—associated with learning outcomes (Goksu and Islam Bolat, 2021). Satisfaction is considered a crucial factor in maintaining learning motivation.

#### 2.1.2 Stimulation strategies in the ARCS motivation model

Keller (1987a) proposed a series of strategies for motivational enhancement based on his ARCS model. Initially, there were 22 teaching strategies concerning the four factors. They were later streamlined into twelve strategies as shown in Table 1 (Cai et al., 2022).

**Table 1:** ARCS model-based teaching strategies.

Dimension	Sub-dimension	Teaching strategies
Attention	Perceptual arousal	Attract students’ interest and arouse curiosity in a novel way.
	Inquiry arousal	Stimulate students’ desire to explore and enhance their curiosity through questions and other means.
	Variability	Attract and maintain interest through diverse teaching methods and activities.
Relevance	Goal orientation	Ensure students understand the practicality and value of learning tasks.
	Motive matching	Provide opportunities and activities that match students’ motivation and learning styles.
	Familiarity	Provide specific examples related to learners’ experiences to enhance their familiarity with learning materials.
Confidence	Learning requirements	Ensure students clearly understand learning requirements and evaluation criteria; Imparting reading skills to help students establish expectations for success.
	Success opportunities	Provide activities to increase the chances of success and enhance students’ belief in their abilities.
	Personal control	Guide students to form appropriate attributions.
Satisfaction	Intrinsic reinforcement	Enable students to apply newly acquired knowledge or skills in real-life situations.
	Extrinsic reward	Motivate students through verbal praise and other positive feedback.
	Equity	Adopt fair examination and evaluation methods and treat students equally.

The dimensions and sub-dimensions, along with their respective teaching strategies, in Table 1 served as a reference for the design of motivational strategies used in the current study’s instructional intervention. In addition, the instructional team tailored the design to suit the specific characteristics of the research subjects in Chinese universities.

### 2.2 Reading motivation

This section utilizes the ARCS model as a guide and systematically analyzes the various forms of its four elements in the English reading motivation of Chinese learners. In the Attention dimension of the ARCS model, Jeon (2021) analyzed the reading motivation of Korean primary and secondary school students. He found that a strong personal interest in reading materials is the main reason individuals choose to read. When students find the content interesting, they naturally pay more attention, which allows them to stay engaged. This research validates that Attention remains a primary driver underlying people’s motivation to participate in reading activities. Accordingly, the choice of interest-tailored reading resources stands out as a pivotal approach to enhance students’ engagement in class (Aukerman and Chambers Schuldt, 2021).

The Relevance dimension refers to the importance of matching instructional content with students’ goals, needs, and values. Fukuda (2024) found that in exam-centered school systems, the primary motivation for Japanese students to read stems from the desire for grades and teacher evaluations. Fukuda’s (2024) findings suggest that educational goals and societal expectations shape the formation of students’ motivation. Shaalan et al. (2023) found through their research that reading content that aligns with learners’ cultural backgrounds significantly increases the reading motivation of these Arabic English learners. All studies emphasize that teachers should tailor their instruction to learners’ experiences and desired goals. Applying teaching strategies based on this principle is crucial for successfully stimulating engagement in language learning (Papi and Khajavy, 2021).

The main goal of the Confidence dimension is to cultivate students’ self-confidence. The Self-Determination Theory posits that students motivated to learn tend to perceive themselves as capable of controlling their behaviors (Ryan et al., 2021). The emphasis of Self-Determination Theory on learners’ perceived autonomy and control is highly consistent with the ARCS model. Duke and Cartwright (2021) also reported that college students who had accumulated reading experiences were more motivated and believed in their reading abilities. Based on the findings, the current study can conclude that providing students with the confidence to succeed encourages them to be more motivated readers, helping them stay more attentive while reading (Acosta-Gonzaga and Ramirez-Arellano, 2022).

When it comes to the Satisfaction dimension, it primarily addresses students’ positive emotions during the learning process. According to Gentilini and Greer (2020), enjoyment-driven reading and experiencing a sense of achievement are key components that help sustain students’ motivation. Zou et al. (2021) found that peer collaboration and pedagogical support, as crucial social factors, significantly influenced the reading practices of Chinese students. Cheng et al. (2021) concluded that a positive classroom culture augments satisfaction levels among students, thus corroborating the role of the Satisfaction dimension in motivation. According to the findings, English reading courses should incorporate team-based activities and interactive components to help learners feel satisfied and take pride in their achievements.

Collectively, research outcomes from studies across diverse cultures have demonstrated that the ARCS model serves as an instrument to boost readers’ motivation and cultivate their long-term interest in reading. For instructors of college English in China, these studies offer well-formulated theories and actionable strategies for integrating the model into their instructional settings. However, educators need to consider both cultural differences and the individual needs of each student when implementing the model (Song and Kao, 2023). By applying the four dimensions of the ARCS model—Attention, Relevance, Confidence, and Satisfaction—teachers can develop corresponding teaching methods to help Chinese college students become more motivated and enjoy reading.

### 2.3 Reading ability

The definition of reading ability involves both theoretical and practical aspects. The China’s Standards of English Language Ability (CSE), issued by the Ministry of Education of the People’s Republic of China (2018), classifies reading ability into different levels, with the descriptors of each level covering information extraction, inference, analysis, and evaluation. These descriptors align with the requirements of the College English Test Band 4 (CET-4), serving as a basis for measuring the English reading proficiency of college students.

In addition, Barnett (1988) explained that reading ability consists of six parts: text comprehension, theme summarization, reasoning, judgment, evaluation, and appreciation. Kim (2016) proposed that reading ability consisted of reading strategies and cognitive abilities. Kim (2016) further subdivided the latter dimension into three components: recognition and extraction, analysis and summarization, and criticism and evaluation.

In this study, we conceptualize reading ability as encompassing four dimensions. Four key sources guided the conceptualization of reading ability: the aforementioned definitions, the Chinese College English Curriculum Standards for reading ability, the reading descriptors in CSE, and the question types in the CET-4 reading comprehension. Table 2 provides the definitions of the four dimensions.

**Table 2:** Classification of reading ability.

Type of reading ability	Definition
Word guessing	Inferring the meaning of unfamiliar words based on background knowledge or context.
Grasping details	Identifying specific information by understanding the literal meanings of words or sentences.
Inference	Inferring deeper meanings by analyzing the author’s attitude and discourse structure.
Theme summarization	Summarizing the main idea and discerning the purpose of the reading material.

### 2.4 Research questions

Regarding the application of the ARCS motivation model, studies are primarily found in the fields of computer-assisted instruction, multimedia teaching, and distance learning (Hu et al., 2016; Li and Ren, 2018; Pribadi et al., 2021; Ritter, 2020). For instance, in flipped classroom settings, the ARCS model has significantly enhanced students’ motivation levels and academic achievement (Ritter, 2020). Li and Moore (2018) introduced the ARCS motivation model in MOOCs to explore how to design effective intervention strategies to enhance learning motivation.

The majority of studies mentioned above focus on high school students (Goksu and Islam Bolat, 2021). However, after passing the highly competitive college entrance examination in China, many university students tend to become disengaged from English courses (Li, 2022). Therefore, it is of great importance to stimulate their motivation for learning English in higher education institutions. Regarding English reading instruction, it plays a vital role in college English courses, but empirical research on its effectiveness is limited (Rezai et al., 2025). Therefore, this study aims to apply the ARCS motivation model to English reading instruction in Chinese universities and investigate its impact on students’ motivation for English reading and proficiency. To achieve the research objective, this study raises the following three questions:

1. What is the impact of ARCS-based English reading instruction on university students’ English reading motivation?
2. What is the impact of ARCS-based English reading instruction on university students’ English reading ability?
3. What is the impact of English reading motivation on university students’ English reading ability?

## 3 Method

### 3.1 Subjects

Based on a quasi-experimental design, this study used two parallel sophomore classes (each comprising 40 students) from a university in Shanghai as samples, dividing them into an experimental group and a control group through random assignment. In terms of teaching variables, the experimental and control groups were comparable in terms of class size, course content, and other factors. All participants voluntarily signed the informed consent form, and the university’s ethics committee formally approved the research plan.

### 3.2 Instruments

#### 3.2.1 English reading motivation scale

The English Reading Motivation Questionnaire developed in this study systematically integrates the core constructs of Keller’s (1987a) Course Interest Survey (CIS) and Aydemir and Öztürk's (2013) Reading Motivation Scale. This study methodically structured the instrument into two distinct sections. The first section gathers participants’ demographic information. The second section, initially comprising 24 items, employs a 5-point Likert scale (1 = Strongly Disagree, 5 = Strongly Agree) for data collection.

To ensure cultural validity and relevance within the Chinese educational context, the research team carefully adapted the items to reflect the specific characteristics of English learning among Chinese university students. For example, in the Attention dimension, the original item in Keller’s scale, “The topics in the course are interesting,” was revised to “I am interested in the themes of English reading materials.” This revision aligns the scale more closely with the thinking habits and linguistic expressions of Chinese learners, while incorporating content related to reading interest from Aydemir’s scale. Item 5 in the Relevance dimension, “English reading is beneficial for my future career development,” is a good example of theoretical integration. The item aligns with Aydemir and Öztürk’s Reading Achievement construct and also resonates with Keller’s Goal Orientation principle. Since reading speed anxiety is a common issue for Chinese college students, the questionnaire includes Item 10, “I have confidence in my English reading speed.” The research team developed this item based on the Task Completion Ability construct from the CIS scale. In the Satisfaction dimension, Item 17 effectively combines Keller’s concept of positive emotions with Aydemir and Öztürk’s approach to measuring satisfaction with English reading. Via the cross-cultural adjustment, the questionnaire in this study maintains its original conceptual framework and fits with the needs of Chinese university learners.

The questionnaire was piloted among 150 Chinese university students to assess its reliability and validity. Participants were selected from comprehensive universities, science and engineering universities, and finance and economics universities to represent a diverse range of subjects and academic levels. There were 136 valid responses in the survey, giving an effective response rate of 90.7% (136/150). Through strict quality control, the research team removed six questionnaires during the data cleaning stage. First, they excluded any questionnaire with more than one-third of its data missing. Second, they eliminated responses that showed unusual patterns, such as selecting the same option for every item. Third, they discarded questionnaires with apparent inconsistencies or signs that the respondent did not take the task seriously.

Exploratory factor analysis (EFA) was used in this study to examine data from the 130 valid questionnaires. This study used principal component analysis (PCA) to identify factors and then applied varimax orthogonal rotation to refine the factor structure. The analysis revealed that the Kaiser-Meyer-Olkin (KMO) measure was 0.909, and Bartlett’s test of sphericity yielded a chi-square value of 1583.26 (*df* = 130, *p* < 0.001), confirming that the data were suitable for factor analysis. This study used a sequence of criteria to screen the items. The researchers excluded items with factor loadings below 0.50, as these items lacked a strong connection with the primary constructs (Petrowski et al., 2021). Additionally, the researchers eliminated items with loading differences of less than 0.20 across dimensions, as they did not fit into the same dimension (Howard, 2023). Third, the researchers removed any items with an item-total correlation coefficient of less than 0.40, indicating that they had a weak link with the overall scale (Shrestha et al., 2023). After screening the original 24 items, the researchers retained 17 with strong discriminative power. The study assigned these items to four dimensions: Attention, Relevance, Confidence, and Satisfaction. According to reliability analysis, the Cronbach’s alpha coefficients ranged from 0.76 to 0.84 for each dimension and 0.89 for the whole scale, indicating consistency at both subscale and overall levels. The researchers excluded a total of seven items: they removed two items from the Attention dimension due to semantic redundancy, eliminated three items from the Relevance dimension because of limited cultural adaptability, and discarded two items from the Confidence dimension due to their low discriminative capacity.

After completing the pretest and revision of the questionnaire, the research team conducted a formal survey using the questionnaire among 611 college students aged 18 and above from five universities. This study adopted a stratified random sampling method to ensure the representativeness of the sample in terms of university types and grade levels. Identity verification measures were employed to prevent repeated participation by the same individual, thereby ensuring the independence and validity of the sample data.

By April 10, 2023, the researchers had completed data collection, obtaining 619 responses with a response rate of 94.9%. To maintain high data quality standards, the researchers implemented a rigorous quality control process, excluding questionnaires that contained incomplete responses, repetitive answers, or conflicting information during analysis. After eliminating eight invalid responses, the researchers retained 611 responses for the next stage of data analysis. The 611 responses were randomly split into two groups to improve both reliability and validity for the research: With the first group (*n* = 300), the study aimed to measure internal consistency, assess structural validity and identify the factor structure of the questionnaire through EFA; The researchers employed the second group (*n* = 311) to perform Confirmatory Factor Analysis (CFA) to confirm the validity of the factor structure previously identified in the EFA. The researchers designed the two-phase analytical process to fulfill two objectives. The first entailed thoroughly evaluating the scale with the combination of exploratory and confirmatory techniques. Additionally, validating the factor structure in different samples strengthened the validity and the generalizability of the instrument. In the reliability assessment phase of the formal questionnaire survey, the scale demonstrated an overall Cronbach’s alpha coefficient of 0.922. Detailed reliability statistics are shown in Table 5 in the Supplementary materials. This statistically rigorous value indicates a strong correlation among the scale items, reflecting high internal consistency. The close interrelationship underpins the stability and reliability of the measurement results for the targeted constructs.

During the EFA, the research team used PCA with varimax rotation. Consistent with the suggestions of Cheung et al. (2024), Fan et al. (2022), and Wang et al. (2023), a rigorous set of criteria was set up to test construct validity. Specifically, the team required that each retained latent factor have an eigenvalue greater than 1, the total variance explained by all the retained factors be greater than 60%, and the loading of each item on its corresponding factor be no lower than 0.50. The researchers systematically excluded items that failed to meet these thresholds from further analysis. As shown in Table 3, the final factor solution comprised four factors, collectively accounting for 66.053% of the total variance. All retained items demonstrated factor loadings that met or exceeded the established criteria, providing strong evidence of the scale’s construct validity. These results support both the theoretical coherence of the scale’s conceptual framework and its practical utility and reliability in empirical research contexts.

**Table 3:** Exploratory factor analysis results.

Factor	Item No. and Description	Factor loading	Initial eigenvalue	% of variance	Cumulative %
Confidence	I8 The English reading textbook is of a moderate level of difficulty.	0.738	7.618	44.811	44.811
	I11 Through hard work, I can improve my English reading ability.	0.710			
	I10 I have confidence in my English reading speed.	0.696			
	I13 My teacher acknowledges my effort in English reading.	0.678			
	I12 I actively participate in class activities related to English reading.	0.672			
	I14 I can effectively apply the reading strategies I have learned to English reading tasks.	0.627			
	I3 I have a clear understanding of the learning objectives in the English reading class.	0.540			
Relevance	I6 I enjoy discussing topics related to English reading textbooks with my classmates.	0.787	1.416	8.329	53.140
	I5 English reading is beneficial for my future career development.	0.692			
	I7 Engaging in English reading can enhance my performance in examinations.	0.653			
	I9 The themes of English reading textbooks are relevant to my daily life.	0.594			
Attention	I2 The questions raised by my teacher in the English reading class are thought-provoking.	0.847	1.172	6.891	60.031
	I1 I am interested in the themes of English reading materials.	0.748			
	I4 The diverse instructional methods used by my teacher enhance my interest in English reading.	0.694			
Satisfaction	I16 I find English reading enjoyable.	0.873	1.024	6.022	66.053
	I17 I am generally satisfied with my performance in English reading tests.	0.848			
	I15 My teacher’s evaluation of my English reading assignments generally aligns with my self-assessment.	0.538			

According to the rotated component matrix presented in Table 3, the analysis grouped the 17 items into four distinct factors. The first factor, labeled Confidence, consisted of seven items: I8, I11, I10, I13, I12, I14, and I3. The second factor, named Relevance, included four items: I6, I5, I7, and I9; the third factor, Attention, comprised three items: I2, I1, and I4. The final factor, Satisfaction, also consisted of three items: I16, I17, and I15.

After conducting reliability and validity analyses, as well as EFA on 300 out of the 611 collected questionnaires, the research team subsequently conducted CFA on the remaining 311 questionnaires. According to Miketinas et al. (2016), CFA—primarily conducted using AMOS—can be divided into two stages. In the present study, the first stage involved assessing the factor loadings of each observed variable on its corresponding latent construct. The second stage focused on evaluating the overall model, which included four latent variables, their corresponding observed indicators, and associated error terms, to determine whether the data supported the model.

The research team employed six model fit indices. The first three were the Goodness-of-Fit Index (GFI), the Adjusted Goodness-of-Fit Index (AGFI), and the Comparative Fit Index (CFI). If the values of these three indices exceeded 0.90, it was considered indicative of good model fit (Alamer and Marsh, 2022). The fourth and fifth indices focused on residuals, assessing model fit based on the magnitude of discrepancy between observed and predicted values; smaller residuals indicated better model-data fit. Specifically, the Root Mean Square Residual (RMR) should be less than 0.10, and the Root Mean Square Error of Approximation (RMSEA) should be below 0.08 (Romm and Alvis, 2022). The final index, CMIN/DF, was considered acceptable when its value was below 5, indicating an adequate model fit (Zisberg et al., 2021).

Regarding the two factors—Attention and Satisfaction—their models were saturated because each contained only three observed variables, resulting in a GFI of 1, the theoretical maximum (Edwards and Konold, 2023). Because the CMIN/DF value was 0 in the saturated models, AGFI, CFI, RMSEA, and RMR values could not be computed (Fuertes and Hurtado, 2017). Furthermore, as saturated models are generally assumed to achieve a perfect fit with the observed data (Raykov et al., 2013), goodness-of-fit testing was deemed unnecessary for the Attention and Satisfaction models.

According to the CFA results for the other two models—Confidence and Relevance—presented in Table 4, all fit indices (CMIN/DF, GFI, AGFI, CFI, RMR, and RMSEA) fell within their recommended thresholds. In both models, all standardized factor loadings exceeded 0.50 and were statistically significant. These results indicated that both models demonstrated satisfactory model fit and psychometric soundness.

**Table 4:** Model fit indices for CFA models (relevance, confidence, and English reading motivation).

Model type	CMIN/DF	*p*	GFI	AGFI	CFI	RMR	RMSEA
Relevance	2.794	0.061	0.991	0.955	0.995	0.014	0.076
Confidence	1.531	0.091	0.981	0.961	0.994	0.018	0.041
Overall motivation model	1.935	0.000	0.927	0.901	0.968	0.031	0.055

Reference thresholds: GFI/AGFI/CFI ≥ 0.90; RMR ≤ 0.10; RMSEA ≤ 0.08; CMIN/DF ≤ 5.

**Figure 1:**
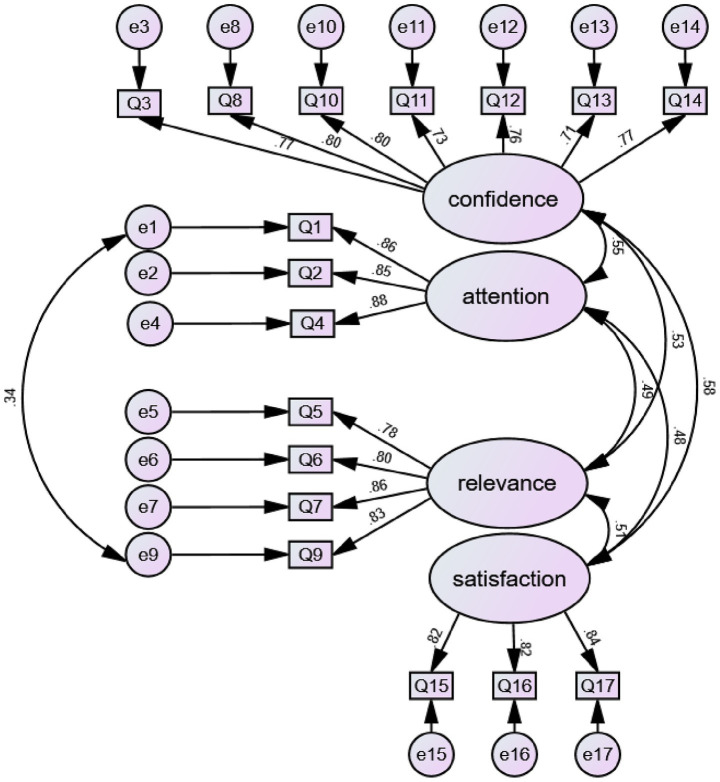
Confirmatory factor analysis of English reading motivation scale.

The final step in the CFA involved evaluating the overall model, which comprised all four latent factors. As shown in Table 4, the fit indices of the modified model fell within the recommended thresholds. Additionally, all standardized regression coefficients, presented in Figure 1, exceeded 0.50. Although the model’s *p*-value was less than 0.05, the large sample size may have contributed to this statistically significant result, despite the model demonstrating an acceptable fit. The complete standardized factor loadings and additional fit statistics are presented in Table 1 in the Supplementary materials. In addition, the correlations among the four latent variables were statistically significant, and all observed variables loaded significantly onto their respective latent constructs. These results support the validity and reliability of the reading motivation model, which comprises the four constructs: Attention, Relevance, Confidence, and Satisfaction.

In the context of scale development and validation, a systematic assessment of reliability and validity is an essential step following CFA. While CFA effectively verifies the construct validity of a measurement model, model fit indices alone are insufficient for a comprehensive evaluation of the scale’s psychometric properties (Le Moine et al., 2016). For this reason, the current study used Cronbach’s alpha coefficient and corrected item-total correlation (CITC) to assess internal consistency (Yeşilyurt, 2024). According to accepted psychometric standards, if the Cronbach’s alpha coefficient exceeds 0.70 or the CITC is greater than 0.40, the scale is considered reliable, and the items statistically reflect the construct (Ariffin et al., 2022).

Construct validity was further assessed through convergent and discriminant validity. The researchers assessed convergent validity using Composite Reliability (CR) and Average Variance Extracted (AVE). A CR value greater than 0.70 indicates satisfactory internal consistency. In contrast, an AVE value greater than 0.50 confirms that the underlying construct explains more than half of the variance in the observed indicators (Dwivedi et al., 2023). The researchers evaluated discriminant validity using the Fornell-Larcker criterion, comparing the square root of the AVE of each construct with its correlation with other constructs to determine its distinctiveness (Ndayizigamiye et al., 2020).

The researchers conducted reliability and validity analyses to ensure measurement consistency and accuracy in representing the construct. This strategy improved the study’s rigor and the validity of its findings.

Psychometric analyses in Table 5 demonstrated the validity and reliability of the scales. Attention, Relevance, Confidence, and Satisfaction all had Cronbach’s alpha coefficients over 0.70, with corresponding values of 0.799, 0.806, 0.864, and 0.857. CITC values ranged from 0.575 to 0.795, indicating good internal consistency. In terms of validity, CR values ranged from 0.866 to 0.908, and AVE values ranged from 0.585 to 0.744, meeting all psychometric benchmarks. Notably, the Attention dimension achieved an AVE of 0.744, suggesting that the latent construct accounted for 74.4% of the variance in its observed indicators.

**Table 5:** Reliability and validity analysis.

Dimension	Measurement indicators	Item	Standardized factor loading	AVE	CR	CITC	Cronbach’s α
Attention	Interest in the theme of the reading material	1	0.857	0.744	0.897	0.623	0.799
	Thought-provoking questions	2	0.85			0.740	
	Interest enhanced by teaching methods	4	0.881			0.575	
Relevance	The professional benefits of reading English	5	0.779	0.667	0.889	0.564	0.806
	Enjoyable topic discussions with classmates	6	0.795			0.705	
	Improvement of examination scores in English reading	7	0.861			0.632	
	Textbooks on topics related to daily life	9	0.83			0.590	
Confidence	Clear learning objectives	3	0.771	0.585	0.908	0.575	0.864
	Textbooks of moderate difficulty	8	0.797			0.626	
	Confidence in reading speed	10	0.802			0.656	
	Achieve an enhancement in reading proficiency through dedicated study	11	0.732			0.699	
	Active participation in classroom activities	12	0.761			0.632	
	The teacher’s recognition of my efforts	13	0.714			0.667	
	The application of learned reading techniques to English reading	14	0.774			0.602	
Satisfaction	Continuous and consistent assessment of reading assignments	15	0.817	0.683	0.866	0.617	0.857
	Love for English reading	16	0.824			0.795	
	Satisfaction with the examination scores of English reading	17	0.838			0.794	

The results of the discriminant validity analysis, presented in Table 6, indicate a strong level of construct separation. The square roots of the AVE for each construct ranged from 0.765 to 0.863. These values were consistently higher than the inter-construct correlation coefficients, which ranged from 0.485 to 0.577. For example, the correlation between the Attention and Satisfaction dimensions was 0.485, which was notably lower than the square root of the AVE for Attention (0.863). This difference supports the presence of adequate discriminant validity among the constructs.

**Table 6:** Discriminant validity: inter-construct correlations and square roots of AVEs.

Dimension	Attention	Relevance	Confidence	Satisfaction
Attention	0.863			
Relevance	0.49	0.817		
Confidence	0.555	0.532	0.765	
Satisfaction	0.485	0.508	0.577	0.826

Diagonal values indicate the square roots of AVEs.

Additionally, all standardized factor loadings ranged from 0.714 to 0.881, falling well within the acceptable range. Taken together, these psychometric indicators suggest that the scale demonstrates strong reliability and validity. These findings confirm the scale’s suitability as a reliable measurement instrument for research in related fields.

So far, the dimensions and their corresponding items of the English Reading Motivation Questionnaire are Attention (Items 1, 2, 4), Relevance (Items 5, 6, 7, 9), Confidence (Items 3, 8, 10, 11, 12, 13, 14), and Satisfaction (Items 15, 16, 17).

#### 3.2.2 Reading test in CET-4

The reading component of CET-4, used in both the pretest and posttest, consists of three sections designed to assess different reading skills: banked cloze (10 items, 10 points), fast reading in a matching format (10 items, 10 points), and intensive reading via multiple-choice questions (10 items, 10 points). Each section targets a specific aspect of reading comprehension, such as vocabulary inference, skimming and scanning, and detailed understanding. The total time allocated for the reading section is 40 min. This structure aligns with the national standards for evaluating university students’ English reading ability in China.

### 3.3 Teaching design

The study adopted the Grammar-Translation method for the control group due to both theoretical and practical considerations. Du (2021) noted that this method remains widely used in college English classrooms in China, with a focus on curriculum teaching for non-English major programs. Under the framework of this method, students focus on grammar learning and master new vocabulary in a systematic way, which brings them two significant advantages: First, it helps teachers easily plan lessons and manage classrooms, enabling them to teach in an organized manner (Syamsia and La Jie, 2024); Second, this method aligns with the requirements of standardized English tests, which mainly assess grammar and vocabulary skills (Juraboyeva et al., 2021). Due to these advantages, it remains a popular approach in practical teaching environments and is widely considered adequate.

To elevate the rigor of the study, this research adopted a structured approach to control extraneous variables in two aspects. On the staff qualification management level, the two teachers assigned to the experimental and the control groups held highly equivalent qualifications. Both instructors possessed a master’s degree in English education and had accumulated at least ten years of teaching experience in tertiary education settings. Their comparable teaching capabilities and styles of classroom management substantially lowered the likelihood of confounding variables related to teaching staff. In terms of process management, both groups used the same course materials and followed a parallel instructional progression. These control measures boosted the internal validity of the study by specifying the teaching method as the exclusive independent variable.

To optimize instructional control, the study introduced a three-phase system consisting of teacher training, teaching implementation, and quality supervision. During the training phase, the two teachers participated in a two-week workshop whose activities covered theoretical learning, simulated teaching sessions, and peer evaluation. As a result, the two teachers developed an accurate understanding of the teaching methods they would employ and their theoretical foundations. The research team also introduced a standardized instructional guidebook during the implementation process. It specified elements such as learning goals, instructional procedures, time allocation, and evaluation criteria. This study required the two teachers to follow the guidebook’s instructions during their teaching practice and to keep daily logs recording lesson progress and student participation. In addition, during weekly debriefing sessions, the research team gained a timely understanding of the implementation of teaching, allowing the two instructors to make necessary adjustments to teaching procedures. At the stage of supervision, the research team implemented a multi-level supervision mechanism. Independent experts, not part of the research team, conducted regular, unplanned classroom observations to assess whether the teaching practice conformed to the standards outlined in the guidebook. Concurrently, the research team interpreted data from teaching records and student feedback. Altogether, these approaches ensured that the teaching practices in both the experimental and control groups strictly conformed to the pre-set protocols, reducing the effect of uncontrollable variables.

The experimental group’s English reading instruction, unlike that of the control group, was guided by the ARCS motivation model. Table 7 shows that this teaching strategy drew on the model’s four components: Attention, Relevance, Confidence, and Satisfaction. To draw students’ attention, the teacher employed a combination of multimedia resources and open-ended questions. For the sake of Relevance, the teacher chose English reading materials that aligned with the students’ experiences and designed language activities with practical value in their academic and daily lives. To help students build confidence, this study designed a series of tasks with gradually increasing difficulty in English reading instruction, providing support at each stage to assist students in accumulating experience and improving their English reading skills. Finally, to enhance satisfaction, students maintained their interest in learning and took pride in their achievements in English reading through positive peer feedback, teacher comments, and formative assessments. All these well-crafted teaching procedures elevated students’ engagement and rendered the intervention more effective.

**Table 7:** Teaching design based on the ARCS model.

Dimension	Subsection	Time for use	Teaching strategy
Attention	Perceptual arousal	Lead-in	Use multimedia resources, including video and audio materials, to capture students’ attention.
	Inquiry arousal	Lead-in	Pose thought-provoking questions to stimulate discussion on topics related to the upcoming reading.
	Variability	While-reading/ Post-reading	Vary instructional methods to suit individual needs, such as adjusting pronunciation and intonation. Introduce activities like storytelling and role-playing to enhance engagement.
Relevance	Goal orientation	Pre-reading	Encourage students to predict the themes of the reading materials and clarify learning objectives.
	Motive matching	While-reading	Set tasks such as skimming and scanning that align with students’ interests and learning needs.
	Familiarity	Pre-reading/ Post-reading	Choose discussion topics that connect to students’ personal experiences and everyday life.
Confidence	Expectation for success	While-reading	Teach reading strategies that help build students’ expectations of success in reading.
	Success opportunities	While-reading	Set moderately challenging tasks to enhance students’ sense of self-efficacy.
	Personal control	While-reading	Guide students to attribute their success to effort and reinforce their belief that success is within their control.
Satisfaction	Intrinsic reinforcement	Post-reading	Provide opportunities for students to showcase their learning outcomes through activities such as debates and other engaging formats.
	Extrinsic reward	Post-reading	Offer opportunities for external recognition through group and teacher evaluations, as well as praise.

### 3.4 Research process

From early September to late December 2023, this study performed a four-month teaching experiment. The experimental group employed the teaching approach based on the ARCS motivational model in their English reading instruction, while the control group maintained the use of the Grammar-Translation method. To maintain comparability, students in both groups used identical English reading materials, and their course arrangements and time allocation remained consistent.

Before the implementation of the intervention, this study conducted two pretests: one was a survey using the English reading motivation scale developed and validated in this study, and the other was the reading section of the 2019 CET-4 real exam. The researchers conducted the two pretests for three reasons: First, to examine if there were significant disparities in English reading motivation between the experimental group and the control group; second, to explore whether there were significant gaps in the English reading performance between the two groups; and third, to collect baseline information for comparative analyses with the results after the instructional intervention.

Following the teaching experiment, this study employed the same English reading motivation scale as the pretest and the reading questions from the 2020 CET-4 as the posttest tools. Posttest completion led to a comparison between the posttest results and the pretest data to examine whether the ARCS model-based teaching approach was statistically significant.

### 3.5 Data collection and analysis

Researchers collected data using the English reading motivation scale developed in this study, as well as the reading modules of the CET-4, administered in 2019 and 2020. For statistical analysis, the researchers processed all the data with SPSS 22.0.

To investigate whether the teaching strategies based on the ARCS motivation model exert a significant impact on college students’ English reading motivation, this study employed independent samples *t*-tests to compare the scores of two groups of college students on the same motivation scale before and after implementing the teaching intervention. Moreover, within-group paired samples *t*-tests were employed to evaluate if there were significant variations in motivation levels.

To explore the effect of teaching strategies based on the ARCS motivation model on college students’ English reading performance, this study used independent samples *t*-tests to compare the scores of the experimental group and the control group in the reading module of CET-4 in China before and after the intervention. Moreover, the researchers conducted within-group paired samples *t*-tests to assess whether there were significant differences in the English reading proficiency of students in each group after the intervention.

Finally, in order to explore how college students’ English reading motivation relates to their English reading performance, this study used SPSS 22.0 to conduct correlation analysis and multiple regression analysis on the scores of each dimension of the motivation questionnaire after the intervention and the scores of the two groups of students in the reading module of CET-4 during the posttest.

## 4 Results

### 4.1 Impact of ARCS-based teaching on English reading motivation (RQ1)

To examine students’ English reading motivation prior to the intervention, the researchers conducted an independent samples *t*-test on the pretest scores from the experimental and control groups. Table 8 presents the results.

**Table 8:** Comparison of English reading motivation between experimental and control groups (pretest and posttest).

Dimension	Group	Pretest Mean (SD)	Posttest Mean (SD)	*t* (post)	*p* (post)	Cohen’s *d* (post)	*t* (within-group)	*p* (within- group)	Cohen’s *d* (within-group)
Attention	Experimental	2.93 (0.72)	3.58 (0.92)	2.271	0.026*	0.508	−4.228	<0.001**	−0.668
	Control	3.05 (0.79)	3.12 (0.92)				−0.459	0.649	−0.073
Relevance	Experimental	2.64 (0.99)	3.42 (0.91)	2.752	0.007**	0.615	−5.141	<0.001**	−0.813
	Control	2.78 (0.77)	2.86 (0.92)				−0.618	0.540	−0.098
Confidence	Experimental	2.91 (1.01)	3.93 (0.73)	4.447	<0.001**	0.994	−6.733	<0.001**	−1.065
	Control	2.96 (0.95)	3.09 (0.95)				−1.231	0.226	−0.195
Satisfaction	Experimental	2.86 (0.91)	3.65 (0.94)	2.621	0.011*	0.586	−4.766	<0.001**	−0.754
	Control	2.99 (0.99)	3.10 (0.94)				−0.597	0.554	−0.094
Total	Experimental	2.83 (0.67)	3.65 (0.69)	3.750	<0.001**	0.839	−8.251	<0.001**	−1.305
	Control	2.94 (0.64)	3.04 (0.75)				−1.694	0.098	−0.268

*p < 0.05, **p < 0.01.

To examine the impact of ARCS-based instruction on students’ English reading motivation, the researchers conducted both between-group and within-group comparisons using independent-samples and paired-samples *t*-tests, respectively. As shown in Table 8, students in the experimental group exhibited a significant increase in their overall motivation scores from pretest (*M* = 2.83, *SD* = 0.67) to posttest (*M* = 3.65, *SD* = 0.69), with a large effect size (Cohen’s *d* = −1.305, *p* < 0.001). In contrast, the control group showed no statistically significant change over time (*p* = 0.098, *d* = −0.268).

Across all four motivational dimensions—Attention, Relevance, Confidence, and Satisfaction—the experimental group outperformed the control group in the posttest. Notably, the researchers observed the most significant gains in the Confidence dimension, where the posttest mean rose sharply to 3.93 (*SD* = 0.73) from a pretest mean of 2.91 (*SD* = 1.01), with a considerable within-group effect size (*d* = −1.065, *p* < 0.001) and a significant between-group difference (*t* = 4.447, *p* < 0.001, *d* = 0.994). Similarly, the researchers detected significant improvements in the Relevance dimension (*p* = 0.007, *d* = 0.615), the Satisfaction dimension (*p* = 0.011, *d* = 0.586), and the Attention dimension (*p* = 0.026, *d* = 0.508), all of which showed medium to large effect sizes.

Conversely, the control group’s motivational scores remained statistically unchanged in all four dimensions, with negligible effect sizes and non-significant *p*-values, indicating that the traditional teaching method had a minimal impact on students’ reading motivation.

These results collectively indicate that ARCS-based instructional design significantly enhanced students’ motivation for English reading across all measured dimensions, particularly in fostering greater confidence and satisfaction. The observed improvements in students’ motivation across all measured dimensions support the pedagogical efficacy of the ARCS model in addressing motivational deficits commonly found in traditional university English classrooms in China.

**Figure 2:**
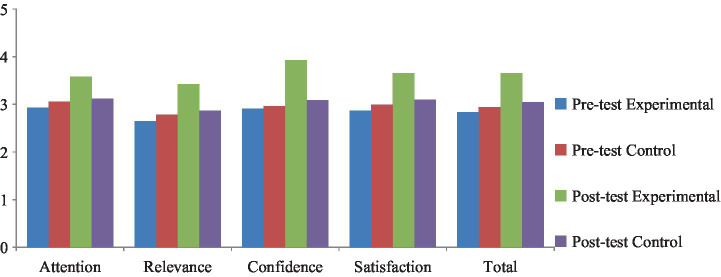
Confirmatory factor analysis of the model—confidence.

To visually supplement the statistical findings presented above, the researchers created a bar chart to illustrate the changes in students’ reading motivation across the ARCS dimensions. As illustrated in Figure 2, the experimental group showed a notable increase in overall English reading motivation, as well as in all four ARCS dimensions, following the intervention. In contrast, the control group showed little to no change across these dimensions. This visual representation reinforces the pattern of improvement observed in the statistical analyses, particularly highlighting the substantial gains in Confidence and Satisfaction among students receiving ARCS-based instruction.

### 4.2 Impact of ARCS-based teaching on English reading performance (RQ2)

To examine whether there were any initial differences in English reading proficiency between the two classes before the intervention, the researchers conducted an independent-samples *t*-test on the pretest scores of the experimental and control classes. According to Table 9, the experimental group and the control group exhibited similar performance in the English reading pretest, with mean scores of 20.78 (*SD* = 2.66) and 20.88 (*SD* = 3.16), respectively. The means of the two groups in the pretest were comparable, suggesting baseline equivalence prior to the intervention. Following the implementation of the ARCS-based instructional approach, the posttest mean score of the experimental group increased significantly to 23.15 (*SD* = 2.90). In contrast, the control group showed only a modest increase to 21.15 (*SD* = 3.03). The between-group difference in the posttest scores was statistically significant (*t* = 3.020, *p* = 0.003), with a medium-to-large effect size (Cohen’s *d* = 0.675), indicating that the ARCS-based instruction effectively enhanced students’ English reading performance.

**Table 9:** Comparison of English reading scores between experimental and control groups (pretest and posttest).

Group	Pretest Mean (SD)	Posttest Mean (SD)	*t* (post)	*p* (post)	Cohen’s *d* (post)	*t* (within-group)	*p* (within-group)	Cohen’s *d* (within-group)
Experimental	20.78 (2.66)	23.15 (2.90)	3.020	0.003**	0.675	−4.855	<0.001**	−0.768
Control	20.88 (3.16)	21.15 (3.03)				−0.531	0.598	−0.084

**p < 0.01.

Within-group comparisons further reinforce this finding. The experimental group demonstrated a statistically significant improvement from the pretest to the posttest (*t* = −4.855, *p* < 0.001), with a large effect size (*d* = −0.768), indicating substantial learning gains. In contrast, the improvement in the control group was not statistically significant (*t* = −0.531, *p* = 0.598), and the effect size was negligible (*d* = −0.084), indicating that traditional instruction had a minimal impact. These results clearly show that the ARCS teaching model is more effective than conventional methods in improving students’ English reading proficiency.

**Figure 3:**
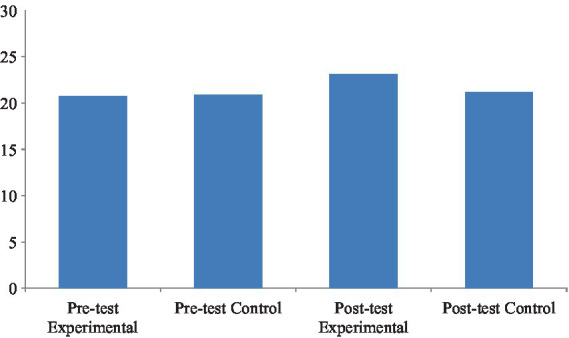
Confirmatory factor analysis of the model—relevance.

To complement the statistical findings, Figure 3 presents a comparison of English reading test scores before and after the intervention for both groups. While both groups started with comparable pretest performance, only the experimental group demonstrated a marked improvement in posttest scores. The control group’s progress was minimal, suggesting that the conventional teaching approach had a limited effect on reading development. This visual pattern aligns with the quantitative findings and further supports the pedagogical effectiveness of the ARCS model in promoting English reading proficiency.

### 4.3 Predictability of motivational factors in English reading proficiency (RQ3)

In this study, the researchers employed multiple linear regression analysis to investigate the predictive value of the distinct dimensions of English reading motivation on reading achievement. Prior to conducting the regression analysis, they performed a Pearson’s correlation analysis to examine the relationships among the variables (as shown in Table 10). The results revealed significant positive correlations between reading achievement and Attention (*r* = 0.398, *p* < 0.01), Relevance (*r* = 0.458, *p* < 0.01), Confidence (*r* = 0.512, *p* < 0.01), and Satisfaction (*r* = 0.574, *p* < 0.01). Additionally, significant positive correlations were found among the various dimensions of motivation (*p* < 0.01). These findings provide essential empirical support for the subsequent regression analysis, enabling a deeper exploration of the mechanisms underlying the relationship between reading motivation and achievement.

**Table 10:** Pearson correlation analyses between dimensions of English reading motivation and posttest reading scores for two student classes.

Variable	Reading test score	Attention	Relevance	Confidence	Satisfaction
Reading test score	1				
Attention	0.398**	1			
Relevance	0.458**	0.627**	1		
Confidence	0.512**	0.470**	0.645**	1	
Satisfaction	0.574**	0.574**	0.573**	0.470**	1

**p < 0.01.

In the present study, the research team constructed a multiple linear regression model with English reading test scores as the dependent variable and Attention, Relevance, Confidence, and Satisfaction as independent variables. The regression analysis, using the Enter method, yielded the results presented in Table 11. The overall model was statistically significant (*F* (4.75) = 12.762, *p* < 0.001), indicating its strong explanatory power. The four motivational dimensions together accounted for 40.5% of the variance in reading scores, as indicated by an *R*^2^ value of 0.405 and an adjusted *R*^2^ value of 0.373. Upon further analysis, Confidence (*β* = 0.298, *p* = 0.014) and Satisfaction (*β* = 0.418, *p* = 0.001) emerged as significant positive predictors of reading scores, highlighting their substantial influence on academic performance. In contrast, Attention and Relevance did not show significant predictive power (*p* > 0.05), suggesting their limited impact on reading achievement within this model. Additionally, collinearity diagnostics revealed that the Variance Inflation Factor (VIF) values ranged from 1.709 to 2.350 for all variables, well below the commonly accepted threshold of 5 (Dormann et al., 2013). The acceptable range of VIF values indicates the absence of multicollinearity, confirming the reliability and validity of the model results.

**Table 11:** Summary of multiple linear regression analyses on the impact of motivational factors on reading test scores.

Variable	Unstandardized Coefficients		Standardized Coefficients	*t*	*p*	Collinearity Statistics	
	*B*	Std. Error	*Beta*			VIF	Tolerance
Constant	13.909	1.249	–	11.137	<0.001**	–	–
Attention	0.005	0.401	0.001	0.012	0.991	1.865	0.536
Relevance	0.083	0.446	0.026	0.187	0.852	2.350	0.426
Confidence	0.984	0.390	0.298	2.522	0.014*	1.763	0.567
Satisfaction	1.337	0.372	0.418	3.591	0.001**	1.709	0.585
*R* ^2^	0.405						
Adjusted *R* ^2^	0.373						
*F*	*F* (4.75)	=12.762, *p* = 0.000					

Dependent Variable: English reading test score. *p < 0.05, **p < 0.01.

## 5 Discussion

### 5.1 Positive effect of ARCS-based teaching approach on English reading motivation (RQ1)

According to the findings of this paper, the use of strategies based on the ARCS model in teaching English reading at universities is likely to increase students’ motivation levels. Positive changes occur because teachers apply the four dimensions of the ARCS model to real teaching scenarios. The instructional design in the Attention dimension keeps students continuously focused; the instructional design in the Relevance dimension helps students perceive the value of reading tasks and actively engage in learning; the teaching support in the Confidence dimension encourages students to mobilize their cognitive resources and conduct in-depth information processing to complete challenging tasks; in terms of the Satisfaction dimension, positive feedback from teachers and peers allows students to experience the joy of learning.

Regarding the Attention dimension, teachers in the experimental group employed methods such as video introductions, interactive discussions, and role-playing to attract students’ attention. These approaches are consistent with the arousal theory (Porges, 2021), which states that diverse external stimuli can mobilize students’ initiative. Meanwhile, the teaching model of the experimental group aligns with the constructivist view of situated learning. Only when teachers provide students with engaging and realistic learning scenarios can students consciously accept reading tasks and maintain their focus for an extended period.

Relevance means that educators should design instruction according to the differences among students. Before class, teachers screen and collect English reading materials related to students’ life experiences by identifying English reading topics; in class, they emphasize the relevance and usefulness of these contents in students’ personal or professional lives. This approach aligns with the Expectancy-Value Theory (Eccles and Wigfield, 2023), which posits that the value of a task serves as the basis for behavioral investment and that both intrinsic and extrinsic aspects express this value. If students find that they can apply what they have learned in real-life situations, their motivation to learn will naturally increase. On the other hand, the traditional Grammar-Translation method teaches mechanical knowledge, ignoring learning styles that connect with authentic tasks and real-life situations. As a result, it hinders the development of a sound understanding of the task’s values (El Morabit and Manegre, 2024). However, the ARCS model can help students break the limitations and make connections between content and objectives.

Regarding Confidence, our study employed a step-by-step approach to developing English reading skills. In the teaching practice of the experimental group, teachers design tasks of moderate difficulty based on students’ cognitive load. As students complete tasks with gradually increasing difficulty, they experience a continuous enhancement in their reading ability and self-confidence. The task design model for reading in the experimental group aligns with the Confidence dimension of the ARCS model and self-efficacy theory (Poluektova et al., 2023). Both emphasize the continuous setting of goals that are challenging yet appropriate, as well as strengthening students’ recognition of the value of learning objectives.

Furthermore, the present study employed two approaches to enhance students’ confidence: firstly, breaking down the overall learning objectives into organized sub-goal modules of progressively increasing difficulty, while ensuring that the difficulty of the English reading task matched the students’ ability. Second, teachers provided timely and appropriate feedback on students’ progress in reading English, helping students to attribute their progress to stable internal factors within their control. Timely feedback is crucial because if teachers fail to provide timely feedback, learners may struggle to recall specific behaviors when reading in English, and may not be able to associate reading success with their efforts or strategies, instead attributing it to external and uncontrollable factors. These two approaches prevent both a decline in motivation levels due to high goal-setting in traditional teaching and stagnation of students’ abilities due to the oversimplification of learning tasks. Thus, this process creates a virtuous circle between increased competence and increased motivation-- boosted motivation contributes to increased competence, which in turn enhances motivation.

To enhance the students’ satisfaction, this study designs a system that integrates multiple positive feedback approaches. It works by organizing individualized showcases of learning outcomes and providing feedback on students’ teamwork performance. By combining teacher evaluations with peer assessment, the system responds to students’ need for external recognition. Meanwhile, it enhances students’ awareness of their academic achievements, thereby strengthening their self-identity in English reading. The instructional design in this study, which focuses on the Satisfaction dimension, follows the principles of Self-Determination Theory (Deci and Ryan, 2000). Fang et al. (2024) proposed that, because the ARCS model is highly operable, it is well-suited for instructional design in terms of both teacher-student interaction and student–student interaction. The interactive educational approach, guided by the ARCS model, can enhance students’ sense of belonging to the class, making them more willing to join a learning community where they help one another. When others recognize and affirm students’ efforts, the students no longer worry excessively about gaining acknowledgment. At the same time, they are more likely to mobilize cognitive resources to take the initiative in learning. Active exploration, in turn, promotes the development of high-quality education while improving emotional and cognitive development.

Additionally, the Chinese cultural background exhibits strong collectivist traits and incorporates key elements of Confucianism. This study investigates the impact of these characteristics on college students’ motivation to read English. Chen et al. (2005) proposed that, in addition to intrinsic desires and achievement goals, cultural influences shape how Chinese students approach English learning. In particular, family members often place high expectations on students, teachers actively provide evaluations that affect students’ motivation, and the broader social environment reinforces the importance of indirect recognition rooted in Confucian “face culture.” For example, when students demonstrate outstanding English reading ability, they may help their parents gain respect among relatives and friends. At the same time, students actively seek to align with group norms to strengthen their social identity. When they raise their English reading ability to meet or exceed the class average, they avoid marginalization and contribute to maintaining class progress, thereby fulfilling collective expectations. Other studies have also shown that excellent exam results and recognition from teachers or others can effectively enhance the enthusiasm for English learning (Bai, 2020; Gao et al., 2014).

Given these special cultural influencing factors, English educators need to make appropriate adjustments when applying the ARCS model. In terms of the Relevance dimension, teachers can capitalize on students’ emphasis on the sense of group belonging and highlight the significance of completing challenging English reading tasks to their families. For example, mastering advanced English reading skills can prepare students for studying abroad in the future and becoming a source of pride for their families. In the Satisfaction dimension, teachers regularly provide parents with feedback on their students’ progress in English reading. Through parents’ recognition, students can feel valued by others and thus develop a sense of belonging to their learning and living environment.

In conclusion, the above analysis reveals that the ARCS model effectively integrates students’ external motivation for English reading (such as English reading scores and teachers’ evaluations) with their internal motivation (including cultivating their interest in English reading and confidence in it). Compared with the Grammar-Translation method, the ARCS model enables students to participate more actively in English reading teaching activities. Therefore, the ARCS model not only possesses strong theoretical and operational properties but can also significantly enhance the teaching effect of English reading classes, providing a more useful reference for the application of motivation theories in English reading classroom activities.

### 5.2 Positive effect of ARCS-based teaching approach on English reading performance (RQ2)

The results of the empirical analyses in this study demonstrate that the ARCS model significantly enhanced the experimental group’s English reading abilities in terms of extracting details, inferring word meanings, reasoning with sentences and paragraphs, and summarizing the main idea of the text. This study concludes that the ARCS model can not only effectively improve students’ English reading skills but also aid in the development of a more scientific and feasible teaching program. This conclusion gains further validity from the researchers’ efforts to ensure that, apart from the teaching method, the experimental and control groups remained consistent in instructional conditions, teaching processes, and students’ English reading proficiency.

Comparing the experimental group to the control group, the former demonstrated a notable improvement in detail extraction. By using visual exercises, the instructor actively engaged the class in the Attention dimension. After teaching a passage about various urban transportation systems, for instance, the instructor requested that the class highlight specific parts in the text using various colors or display the information in a visually enhanced format. At the teacher’s request, students marked specific information about buses, subways, or other urban public transportation in London, Tokyo, and New York using highlighters of various colors. These details included operating hours, the number of central lines, departure intervals, fare policies, the results of passenger satisfaction surveys, the history of the lines, and the coverage of the lines. After marking them with various colors, the students organized these details into a comparison table. Due to this approach, students were more attentive to the article, locating the details more quickly, and minimizing confusion that might occur when dealing with occasionally excessive details.

To enhance the experimental group’s vocabulary inference skills, the teacher incorporated the Attention dimension of the ARCS model when designing the vocabulary teaching portion and included contextual clues in the text to increase students’ interest in and attentiveness to vocabulary meanings, thereby enhancing the effect of inferring word meanings. Additionally, from the Relevance perspective, the teacher chose texts closely related to the students’ life experiences, allowing them to apply their prior knowledge to the vocabulary learning process and preventing them from being passive recipients during vocabulary acquisition. As the theme of the article was closely related to their lives, students could also use this vocabulary in similar real-life situations after actively exploring the meanings of the words, which strengthened not only their inferencing skills but also their ability to use unfamiliar vocabulary. In the Satisfaction dimension, the teacher provided students with timely, positive comments and targeted guidance at the right time. The teacher’s accurate and encouraging feedback style increased the experimental group’s self-esteem and sense of accomplishment while also providing the opportunity for the students to engage in more challenging word-meaning inference tasks actively. Lastly, regarding the Confidence dimension, students were more likely to provide accurate attributions when they employed logical inference techniques rather than relying on rote memorization to achieve successful outcomes. Instead of crediting their success in learning vocabulary and deducing the meaning of new words to external or uncontrollable causes, they attributed it to controllable internal ones, including the correct use of reading strategies. Once this logical and optimistic attribution style alleviated anxiety, students could concentrate more on contextual cues and make progress in word meaning inference.

Due to the educational techniques based on the three aspects of Attention, Relevance, and Confidence, students in the experimental group demonstrated a considerable improvement in their inferential abilities at both the sentence and paragraph levels. To provide students with a cognitively supportive framework for the subsequent high-level inferential reading, teachers employed predictive questioning to activate their prior knowledge related to the new text and to capture their attention before they began reading. In terms of reading materials, the teacher selected texts closely related to the students’ life experiences, which helped them connect written content with their background knowledge and infer implicit meanings between the lines during the reading process. In reading instruction, the teacher guided students to use inferential strategies such as analyzing logical relationships, paragraph organization, overall text structure, and making contextual associations to enhance reading comprehension. After students systematically mastered these reading strategies, the enhanced confidence motivated them to engage more actively with advanced English texts. It continued to improve their inferential reasoning skills through the process of higher-level reading, thereby creating a virtuous cycle.

Students in the experimental class also improved their ability to summarize the text’s key points after the introduction of instructional strategies based on the four aspects of the ARCS model. Teachers used mind maps and other visual aids to help guide reading instruction in the Attention dimension. For instance, the visualizing keywords could draw students’ attention based on the features of images’ immediacy and clarity, and the visualization of the text’s logical structure aided students in understanding the flow of ideas to determine its main points. In Relevance, the teacher chose texts with themes pertinent to students’ needs and daily lives. The use of thematically relevant texts enabled them to deconstruct and better understand the comparatively abstract core ideas of the texts by drawing on their own life experiences. In Confidence, the teacher in the experimental group employed a scaffolded approach to develop students’ ability to summarize texts gradually. Firstly, the teacher guided students to identify the main idea of a single paragraph using keywords and topic sentences, while also fostering their ability to synthesize fragmented information. Next, the teacher helped students summarize multiple paragraphs by analyzing logical relationships, such as parallelism, causality, and contrast. Finally, the teacher trained the students to summarize the main idea of an entire passage by applying the reading strategies they had previously learned. With the help of this teaching approach, students improved their self-efficacy and English reading abilities sustainably. Regarding Satisfaction, the teacher in the experimental group provided positive, precise, and timely feedback on students’ effective performance in summarizing main ideas, praising their ability to identify key points and clarify the author’s line of reasoning. Students felt valued and affirmed when their efforts were acknowledged, which gave them a sense of accomplishment and boosted their desire to learn. If students had difficulties in understanding the main ideas of an English article after class, they tended to adjust their strategies on their own, for example, selecting appropriate summarizing methods based on the specific question at hand. Once they were skilled in a range of techniques, they were more likely to shift from passive learners of the textbook content to active constructors of meaning.

In contrast, under the traditional Grammar-Translation method, the teacher in the control group did not activate the students’ background knowledge by connecting the reading materials to familiar contexts. The lack of background knowledge activation caused them to retain a sense of unfamiliarity toward the texts and to devote excessive cognitive resources to understanding relatively abstract and unfamiliar concepts. That left them with insufficient capacity to analyze the logical structure of the discourse, and the increased cognitive load made it more difficult for them to summarize the main ideas of the texts.

As a result, the ARCS model enhances the reading proficiency of college students in English (Romli et al., 2024). It also helps them to continuously improve their reading skills and develop their sense of self-regulation by reflecting on their learning experiences after class (Karaali, 2015).

The ARCS model is one of the more successful teaching models for college English when compared to the Grammar-Translation method and general motivational strategies. It enhances college students’ performance by establishing a well-structured framework for instruction, creating realistic contexts, and providing students with prompt and constructive feedback, all of which significantly improve their reading proficiency.

### 5.3 Predictability of confidence and satisfaction in English reading performance (RQ3)

The study’s regression analysis revealed that, of the four variables in the ARCS motivation model, Confidence and Satisfaction functioned as significant predictors of students’ English reading performance. This finding is crucial for both encouraging students to read in English and improving their academic performance.

Students can utilize their reading and learning skills more effectively if they possess a high level of self-efficacy (David et al., 2024). According to attribution theory (Weiner, 1985), self-assured students often assume that the reasons behind their reading challenges are within their control, such as a lack of vocabulary, insufficient grammatical knowledge to accurately and efficiently analyze complex English sentences, or a misuse of reading strategies. Consequently, this is a type of positive attribution for these students. Confident students are more likely to maintain their focus and make constant adjustments to their reading strategies when presented with challenging reading assignments (Needles and Abramson, 1990). For instance, individuals might attempt different strategies, such as title analysis or mind mapping, to determine the primary theme if high-frequency summarizing techniques are ineffective in helping them understand a passage’s main idea.

Besides, more self-assured students are better able to regulate their emotional states (Jin et al., 2022), which indirectly supports the notion that emotional regulation contributes to cognitive performance (De Pascalis and Moret-Tatay, 2023). When students with high self-evaluations encounter reading difficulties, they are better able to resist the impact of negative emotions during high-level reading tasks. Students with high self-evaluations gain this advantage because they tend to adopt more positive coping strategies, such as cognitive reappraisal and self-talk, than those with low self-evaluations.

Notably, Satisfaction also played a significant predictive role in English reading performance (*β* = 0.418, *p* < 0.01), which aligns with the claims of goal-setting theory (Cheng, 2023). In other words, if students feel delighted after completing a reading task, this sense of satisfaction can enhance their self-efficacy and lead them to set higher reading goals (Cai and Xing, 2025). Once students achieve these higher goals, they experience a corresponding sense of personal accomplishment, which in turn fosters deeper motivation and promotes the continuous improvement of their English reading ability (Foulk et al., 2019).

Based on the data analysis results, this study approaches the enhancement of students’ English reading performance and satisfaction from three aspects. First, moderately challenging texts with an average accuracy rate of 85 to 90% should be provided (Juel, 1996). The second is to increase the emphasis on formative assessment throughout the entire teaching process. For example, teachers assess students’ progress through methods such as learning portfolios, peer assessment, self-evaluation, and visual progress bars. Ultimately, teachers evaluate students’ English reading proficiency through summative assessments, such as final exams. Third, students form learning communities by voluntarily organizing groups outside of the classroom based on mutually agreed-upon rules. Within each group, students assume distinct roles while learning to communicate, listen to others’ perspectives, and share their ideas through collaboration, thereby fostering a positive learning environment (Farmer and Cadwallader, 2000). As a result, students feel a sense of belonging and are more inclined to dedicate themselves to reading assignments both within and outside of the classroom. The task design, formative assessment system, and learning community discussed above foster students’ positive attitudes. These elements help students succeed and demonstrate their ability to complete challenging reading tasks, which increases their willingness to strive toward higher goals, thus further raising their English reading proficiency.

### 5.4 The effect of sample size and sample source on the generalizability of research findings

Although this study employed rigorous experimental design and statistical analysis, the researchers acknowledge certain limitations related to sample selection. Specifically, the sample consisted of 80 sophomore students from a single university, which may constrain the generalizability of the findings in several ways.

First, the relatively small sample size may reduce statistical power, thereby affecting the precision and stability of the estimated effects. Second, the homogeneity of the sample, particularly in terms of cultural background, academic characteristics, and access to educational resources, limits the applicability of the results to broader populations. These factors suggest that researchers should exercise caution when extending the findings to students from different regions, academic programs, or educational levels.

To enhance external validity in future research, researchers should increase the sample size and diversity of their participants. Recruiting participants from multiple institutions, regions, and educational contexts could improve the representativeness of the sample and support broader generalization of the results. Collaborative, multi-site studies may be especially valuable in capturing the variability present in larger educational systems.

## 6 Implications of ARCS-based instructional interventions for college English reading

Situated within the context of Chinese college English classrooms, this study employed a quasi-experimental design to systematically examine the effects of instructional interventions grounded in the ARCS motivation model on students’ English reading motivation and performance. In comparison to the conventional Grammar-Translation method, the ARCS model has markedly enhanced students’ motivation for English reading. Teaching techniques based on this model can fully stimulate their enthusiasm and foster a strong interest in English reading. Raising motivation contributes to improved English reading proficiency, as evidenced by the experimental group outperforming the control group in posttest scores, with a statistically significant difference.

It is important to note that, of the four dimensions mentioned above, Confidence and Satisfaction are the significant predictors of English reading proficiency. Accordingly, the present study suggests that when using the ARCS model, university English reading instruction should emphasize enhancing students’ confidence and sense of achievement.

Although the majority of factors unrelated to teaching strategies—such as class size, teaching materials, and instructors’ level of teaching proficiency—were kept consistent in this teaching experiment, the researchers did not control some confounding variables, including students’ college entrance English scores, family economic background, and parents’ education levels. Therefore, these factors might affect the generalizability of the experiment’s findings.

This study offers two recommendations to guarantee the methodological rigor of subsequent pertinent research. First, researchers should aim to collect comprehensive baseline data from participants during the pretest phase. Subsequent analyses can include potential confounding variables as covariates in statistical models, using methods such as Analysis of Covariance (ANCOVA) or Structural Equation Modeling (SEM), thereby minimizing their impact on the study’s findings. Second, researchers can enhance the extrinsic validity and generalizability of the research findings by broadening the sample to include individuals from various geographic locations, academic majors, and levels of English proficiency. In pursuit of generalizability, cross-regional and cross-institutional surveys hold greater advantages.

In conclusion, this study examined the impact of applying the ARCS model to college English reading instruction through questionnaires and teaching experiments, demonstrating the rationality of combining the two, despite certain areas for improvement regarding variable control and sample representativeness. These findings suggest that designing English reading instruction based on the ARCS model is both necessary and beneficial. Therefore, this study proposes the following recommendations: First, instructors should implement English reading instruction using a multifaceted instructional framework. Second, it should aim to draw and hold students’ attention, establish and emphasize the relevance between instruction and students’ needs, and increase their confidence and sense of satisfaction.

## Data Availability

The original contributions presented in the study are included in the article/Supplementary material, further inquiries can be directed to the corresponding author.
