# Supplementary material for: Enhancing English reading motivation and performance via the ARCS model: an empirical study using the ARCS motivation scale
Source: Front Psychol. 2025 Oct 28;16:1499957. doi: 10.3389/fpsyg.2025.1499957 (PMC12602433; doi:10.3389/fpsyg.2025.1499957)
Supplement: Supplementary file 1 [file Data_Sheet_1.doc]

**English Reading Post-test (40 minutes)**

**Section A (1x10 points)**

**Directions:** In this section, there is a passage with ten blanks. You are required to select one word for each blank from a list of choices given in a word bank following the passage. Read the passage through carefully before making your choices. Each choice in the bank is identified by a letter. Please mark the corresponding letter for each item on your Answer Sheet with a single line through the centre. You may not use any of the words in the bank more than once．

Just because they can’t sing opera or ride a bicycle doesn’t mean that animals don’t have culture. There’s no better example of this than killer whales. As one of the most __1__ predators，killer whales may not fit the __2__ of a cultured creature. However, these beasts of the sea do display a vast range of highly __3__ behaviors that appear to be driving their genetic development.

The word “culture” comes from the Latin “colere,” which __4__ means “to cultivate.” In other words, it refers to anything that is __5__ or learnt, rather than instinctive or natural. Among human populations, culture not only affects the way we live, but also writes itself into our genes, affecting who we are. For instance, having spent many generations hunting the fat marine mammals of the Arctic, the Eskimos of Greenland have developed certain genetic __6__ that help them digest and utilize this fat-rich diet, thereby allowing them to __7__ in their cold climate.

Like humans, killer whales have colonized a range of different __8__ across the globe, occupying every ocean basin on the planet, with an empire that __9__ from pole to pole. As such, different populations of killer whales have had to learn different hunting techniques in order to gain the upper hand over their local prey. This, in turn, has a major effect on their diet, leading scientists to __10__ that the ability to learn population-specific hunting methods could be driving the animals’ genetic development.

| A) acquired | B) adaptations | C) brutal | D) deliberately | E) expressed |
| --- | --- | --- | --- | --- |
| F) extends | G) habitats | H) humble | I) image | J) literally |
| K) refined | L) revolves | M) speculate | N) structure | O) thrive |

**Section B (1x10 points)**

**Directions:** In this section, you are going to read a passage with ten statements attached to each statement contains information given in one of the paragraphs. Identify the paragraph from which the information is derived. You may choose a paragraph more than once. Each paragraph is marked with a letter. Answer the questions by marking the corresponding letter on your Answer Sheet.

**Living with parents edges out other living arrangements for 18-to 34-year-olds**

A) Broad demographic shifts is marital status, educational attainment and employment have transformed the way young adults in the U.S. are living, and a new Pew Research Center analysis highlights the implications of these changes for the most basic element of their lives—where they call home. In 2014,for the first time in more than 130 years, adults ages 18 to 34 were slightly more likely to be living in their parents’ home than they were to be living with a spouse or partner in their own household.

B) This turn of events is fueled primarily by the dramatic drop in the share of young Americans who are choosing to settle down romantically before age 35. Dating back to 1880, the most common living arrangement among young adults has been living with a romantic partner, whether a spouse or a significant other. This type of arrangement peaked around 1960, when 62% of the nation’s 18-to 34-year-olds were living with a spouse or partner in their own household, and only one-in-five were living with their parents.

C) By 2014, 31.6% of young adults were living with a spouse or partner in their own household, below the share living in the home of their parent(s) (32.1%). Some 14% of young adults lived alone, were a single parent or lived with one or more roommates. The remaining 22% lived in the home of another family member (such as a grandparent, in-law or sibling, a non-relative, or in group quarters like college dormitories.

D) It’s worth noting that the overall share of young adults living with their parents was not at a record high in 2014. This arrangement peaked around 1940, when about 35% of the nation’s 18-to 34-year-olds lived with mom and/or dad (compared with 32% in 2014). What has changed, instead, is the relative share adopting different ways of living in early adulthood, with the decline of romantic coupling pushing living at home to the top of a much less uniform list of living arrangements.

Among young adults, living arrangements differ significantly by gender. For men ages 18 to 34, living at home with mom and/or dad has been the dominant living arrangement since 2009, In 2014,28% of young men were living with a spouse of partner in their own home, while 35% were living in the home of their parent(s). Young women, however, are still more likely to be living with a spouse of romantic partner(35%) than they are to be living with their parent(s)(29%).

F) In 2014, more young women (16%) than young men (13%) were heading up a household without a spouse or partner. This is mainly because women are more likely than men to be sigle parents living with their children. For their part, young men (25%) are more likely than young women (19%) to be living in the home of another family member, a non-relative or in some type of group quarters.

G) A variety of factors contribute to the long-run increase in the share of young. Adults living with the parents. The first in the postponement of, if not retreat from, marriage. The average age of first marriage has risen steadily for decades. In addition, a growing share of young adult may be avoiding marriage altogether. A previous Pew Research Center analysis projected that as many as one-in-four of today’s young adult may never marry. While cohabitation has been on the rise, the overall share of young adults either married or living with an unmarried partner has substantially fallen since 1990.

H) In addition, trends in both employment status and wages have likely contributed to the growing share of young adults who are living in the home of their parent(s), and this is especially true of young men. Employed young men are much less likely to live at home than young men without a job, and employment among young men has fallen significantly in recent decades. The share of young men with jobs peaked around 1960 at 84%. In 2014, only 71% of 18-to-34-year-old men were employed. Similarly with earnings, young men’s wages (after adjusting for inflation) have been on a downward trajectory since 1970 and fell significantly from 2000 to 2010. As wages have fallen, the share of young men living in the home of their parent(s) has risen.

I) Economic factors seem to explain less of why young adult women are increasingly likely to live at home. Generally, young women have had growing success in the paid labor market since 1960 and hence might increasingly be expected to be a be to afford to afford to live independently of their parents. For women, delayed marriage—which is related, in part, to labor market outcomes for men—may explain more of the increase in their living in the family home.

J) The Great Recession (and modest recovery) has also been associated with an increase in young adults living at home. Initially in the wake of the recession, college enrollments expanded, boosting the ranks of young adults living at home. And given the weak job opportunities facing young adults, living at home was part of the private safety net help young adults to weather the economic storm.

K) Beyond gender, young adult’s living arrangements differ considerable by education—which is tied to financial means. For young adults without a bachelor’s degree, as of 2008 living at home with their parents was more prevalent than living with a romantic partner. By 2014, 36% of 18-to 34-year-olds who had not completed a bachelor’s degree were living with their parent(s) while 27% were living with a spouse or partner. Among college graduates, in 2014 46% were married or living with a partner, and only 19% were living with their parent(s). Young adults with a college degree have fared much better in the labor market than their less-educated counterparts, which has in turn made it easier to establish their own households.

11. Unemployed young men are more likely to live with their parents than the employed.

12. In 2014, the percentage of men aged 18 to 34 living with their parents was greater than that of their female counterparts.

13. The percentage of young people who are married or live with a partner has greatly decreased in the past three decades or so.

14. Around the mid-20th century, only 20 percent of 18- to 34-year-old lived in their parents’ home.

15. Young adults with a college degree found it easier to live independently of their parents.

16. Young men are less likely to end up as single parents than young women.

17. More young adult women live with their parents than before due to delayed marriage.

18. The percentage of young men who live with their parents has grown due to their decreased pay in recent decades.

19. The rise in the number of college students made more young adults live with their parents.

20. One reason for young adults to live with their parents is that get married late or stay single all their lives.

**Section C (1x10 points)**

**Directions:** There are 2 passages in this section. Each passage is followed by some questions or unfinished statements. For each of them there are four choices marked A), B), C) and D). You should decide on the best choice and mark the corresponding letter on your Answer Sheet with a single line through the centre.

**Passage One**

Questions 21 to 25 are based on the following passage.

According to the majority of Americans, women are every bit as capable of being good political leaders as men. The same can be said of their ability to dominate the corporate boardroom. And according to a new Pew Research Center survey on women and leadership, most Americans find women indistinguishable from men on key leadership traits such as intelligence and capacity for innovation, with many saying they’re stronger than men in terms of being passionate and organized leaders.

So why, then, are women in short supply at the top of government and business in the United States? According to the public, at least, it’s not that they lack toughness, management talent or proper skill sets.

It’s also not all about work-life balance. Although economic research and previous survey findings have shown that career interruptions related to motherhood may make it harder for women to advance in their careers and compete for top executive jobs, relatively few adults in the recent survey point to this as a key barrier for women seeking leadership roles. Only about one-in-five say women’s family responsibilities are a major reason why there aren’t more females in top leadership positions in business and politics.

Instead, topping the list of reasons, about four-in-ten Americans point to a double standard for women seeking to climb to the highest levels of either politics or business, where they have to do more than their male counterparts to prove themselves. Similar shares say the electorate and corporate America are just not ready to put more women in top leadership positions.

As a result, the public is divided about whether the imbalance in corporate America will change in the foreseeable future, even though women have made major advances in the workplace. While 53% believe men will continue to hold more top executive positions in business in the future, 44% say it’s only a matter of time before as many women are in top executive positions as men. Americans are less doubtful when it comes to politics: 73% expect to see a female president in their lifetime.

**21. What do most Americans think of women leaders according to a new Pew Research Center survey?**

A) They have to do more to distinguish themselves.

B) They have to strive harder to win their positions.

C) They are stronger than men in terms of willpower.

D) They are just as intelligent and innovative as men.

**22. What do we learn from previous survey findings about women seeking leadership roles?**

A) They have unconquerable difficulties on their way to success.

B) They are lacking in confidence when competing with men.

C) Their failures may have something to do with family duties.

D) Relatively few are hindered in their career advancement.

**23. What is the primary factor keeping women from taking top leadership positions according to the recent survey?**

A) Personality traits.

B) Family responsibilities.

C) Gender bias.

D) Lack of vacancies.

**24. What does the passage say about corporate America in the near future?**

A) More and more women will sit in the boardroom.

B) Gender imbalance in leadership is likely to change.

C) The public is undecided about whether women will make good leaders.

D) People have opposing opinions as to whether it will have more women leaders.

**25. What do most Americans expect to see soon on America’s political stage?**

A) A woman in the highest position of governmen.

B) More and more women actively engaged in politics.

C) A majority of women voting for a female president.

D) As many women in top government positions as men.

**Passage Two**

**Questions 26 to 30 are based on the following passage.**

People have grown taller over the last century, with South Korean women shooting up by more than 20cm on average, and Iranian men gaining. 16.5cm. A global study looked at the average height of 18-year-olds in 200 countries 1914 and 2014.

The results reveal that while Swedes were the tallest people in the world in 1914, Dutch men have risen from 12th place to claim top spot with an average height of 182.5cm. Larvian women. Meanwhile, rose from 28th place in 1914 to become the tallest in the world a century later, with an average height of 169.8cm.

James Bentham, a co-author of the research from Imperial College, London, says the global trend is likely to be due primarily to improvements in nutrition and healthcare. “An individual’s genetics has a big influence on their height, but once you average over whole populations, genetics plays a less key role,” he added.

A little extra height brings a number of advantages, says Elio Riboli of Imperial College. “Being taller is associated with longer life expectancy,” he said. “This is largely due to a lower risk of dying of cardiovascular disease among taller people.”

But while height has increased around the world, the trend in many countries of north and sub-Saharan Africa causes concern, says Riboli. While height increased in Uganda and Niger during the early 20th century, the trend has reversed in recent years, with height decreasing among 18-year-olds.

“One reason for these decreases in height is the economic situation in the 1980s,” said Alexander Moradi of the Universith of Sussex. The nutritional and health crises that followed the policy of structural adjustment, he says, led to many children and teenagers failing to reach their full potential in terms of height.

Bentham believe the global trend of increasing height has important implications. “How tall we are now is strongly influenced by the environment we grew up in,” he said. “If we give children the best possible start in life now, they will be healthier and more productive for decades to come.”

**26. What does the global study tell us about people’s height in the last hundred years?**

A) There is a remarkable difference across continents.

B) There has been a marked increase in most countries.

C) The increase in people’s height has been quickening.

D) The increase in women’s height is bigger than in men’s.

**27. What does James Bentham say about genetics in the increase of people’s height?**

A) It counts less than generally thought.

B) It outweighs nutrition and healthcare.

C) It impacts more on an individual than on population.

D) It plays a more significant role in females than in males.

**28. What does Elio Riboli say about taller people?**

A) They tend to live longer.

B) They enjoy an easier life.

C) They generally risk fewer fatal diseases.

D) They have greater expectations in life.

**29. What do we learn about 18-year-olds in Uganda and Niger?**

A) They grow up slower than their peers in other countries.

B) They are actually shorter than their earlier generations.

C) They find it hard to bring their potential into full play.

D) They have experienced many changes of government

**30. What does James Bentham suggest we do?**

A) Watch closely the global trend in children’s development.

B) Make sure that our children grow up to their full height.

C) Try every means possible to improve our environment.

D) Ensure our children grow up in an ideal environment.
