# Supplementary material for: Enhancing English reading motivation and performance via the ARCS model: an empirical study using the ARCS motivation scale
Source: Front Psychol. 2025 Oct 28;16:1499957. doi: 10.3389/fpsyg.2025.1499957 (PMC12602433; doi:10.3389/fpsyg.2025.1499957)
Supplement: Supplementary file 2 [file Data_Sheet_2.doc]

**English Reading Pretest (40 minutes)**

**Section A (1x10 points)**

**Directions:** In this section, there is a passage with ten blanks. You are required to select one word for each blank from a list of choices given in a word bank following the passage. Read the passage through carefully before making your choices. Each choice in the bank is identified by a letter. Please mark the corresponding letter for each item on your Answer Sheet with a single line through the centre. You may not use any of the words in the bank more than once．

Ships are often sunk in order to create underwater reefs perfect for scuba diving and preserving marine  1   Turkish authorities have just sunk something a little different than a ship, and it wouldn’t normally ever touch water, an Airbus A300. The hollowed-out A300 was  2   of everything potentially harmful to the environment and sunk off the Aegean coast today. Not only will the sunken plane  3   the perfect skeleton for artificial reef growth, tut authorities hope this new underwater attraction will bring tourists to the area.

The plane    4   a total length of 54 meters, where experienced scuba divers will    5   be able to venture through the cabin and around the plane’s    6   . Aydin Municipality bought the plane from a private company for just under US$100,000, but they hope to see a return on that    7   through the tourism industry. Tourism throughout Turkey is expected to fall this year as the country has been the    8   of several deadly terrorist attacks. As far as sunken planes go, this Airbus A300 is the largest    9   sunk aircraft ever.

Taking a trip underwater and    10   the inside of a sunken A300 would be quite an adventure, and that is exactly what Turkish authorities are hoping this attraction will make people think. Drawing in adventure seekers and experienced divers, this new artificial Airbus reef will be a scuba diver’s paradise.

| A) create | B) depressed | C) eventually | D) experiences | E) exploring |
| --- | --- | --- | --- | --- |
| F) exterior | G) habitats | H) innovate | I) intentionally | J)investment |
| K) revealing | L) stretches | M) stripped | N) territory | O) victim |

**Section B (1x10 points)**

**Directions:** In this section, you are going to read a passage with ten statements attached to each statement contains information given in one of the paragraphs. Identify the paragraph from which the information is derived. You may choose a paragraph more than once. Each paragraph is marked with a letter. Answer the questions by marking the corresponding letter on your Answer Sheet.

**Make Stuff, Fail, And Learn While You’re At It**

A) We’ve always been a hands-on, do-it-yourself kind of nation. Ben Franklin, one of America’s founding fathers, didn’t just invent the lightning rod. His creations include glasses, innovative stoves and more.

B) Franklin, who was largely self-taught, may have been a genius, but he wasn’t really an exception when it comes to American making and creativity.

C) The personal computing revolution and philosophy of disruptive innovation of Silicon Valley grew, in part, out of the creations of the Homebrew Computer Club, Which was founded in a garage in Menlo Park, California, in the mid-1970s. Members — including guys named Jobs and Wozniak — started making and inventing things they couldn’t buy.

D) So it’s no surprise that the Maker Movement today is thriving in communities and some schools across America. Making is available to ordinary people who aren’t tied to big companies, big defense labs or research universities. The maker philosophy echoes old ideas advocated by John Dewey, Montessori, and even ancient Greek philosophers, as we pointed out recently.

E) These maker spaces are often outside of classrooms, and are serving an important educational function. The Maker Movement is rediscovering learning by doing, which is Dewey’s phrase from 100 years ago. We are rediscovering Dewey and Montessori and a lot of the practices that they pioneered that have been forgotten or at least put aside. A maker space is a place which can be in a school, but it doesn’t look like a classroom. It can be in a library. It can be out in the community. It has tools and materials. It’s a place where you get to make things based on your interest and on what you’re learning to do.

F) Ideas about learning by doing have struggled to become mainstream educationally, despite being old concepts from Dewey and Montessori, Plato and Aristotle, and in the American Contcxt, Ralph Emerson, on the value of experience and self-reliance. It’s not necessarily an efficient way to learn. We learn, in a sense, by trial and error. Learning from experience is something that takes time and patience. It’s very individualized. If your goal is to have standardized approaches to learning, where everybody learns the same thing at the same time in the same way, then learning by doing doesn’t really fit that mold anymore. It’s not the world of textbooks. It’s not the world of testing.

G）Learning by doing may not be efficient, but it is effective. Project-based learning has grown in popularity with teachers and administrators. However, project-based learning is not making. Although there is a connection, there is also a distinction. The difference lies in whether the project is in a sense defined and developed by the student or whether it’s assigned by a teacher. We’ll all get the kids to build a small boat. We are all going to learn about X, Y, and Z. That tends to be one form of project-based learning.

H）I really believe the core idea of making is to have an idea within your head — or you just borrow it from someone — and begin to develop it , repeat it and improve it. Then, realize that idea somehow. That thing that you make is valuable to you and you can share it with others. I’m interested in how these things are expressions of that person, their ideas, and their interactions with the world.

I）In some ways, a lot of forms of making in school trivialize（使变得无足轻重）making. The thing that you make has no value to you. Once you are done demonstrating whatever concept was in the textbook, you throw away the pipe cleaners, the cardboard tubes.

J）Making should be student-directed and student-led, otherwise it’s boring. It doesn’t have the motivation of the student. I’m not saying that students should not learn concepts or not learn skills. They do. But to really harness their motivation is to build upon their interest. It’s to let them be in control and to drive the car.

K）Teachers should aim to build a supportive, creative environment for students to do this work. A very social environment, where they are learning from each other. When they have a problem, it isn’t the teacher necessarily coming in to solve it. They are responsible for working through that problem. It might be they have to talk to other students in the class to help get an answer.

L) The teacher’s role is more of a coach or observer. Sometimes, to people, it sounds like this is a diminished rote for teachers. I think it’s a heightened role. You’re ereating this environment, like a maker space. You have 20 kids doing different things. You are watching them and really it’s the human behaviors you’re looking at . Are they engaged? A they developing and repeating their project? Are they stumbling (受挫)？ Do they need something that they don’t have? Can you help them be aware of where they are?

M)My belief is that the goal of making is not to get every kid to be hands-on, but it enable us to be good learners. It’s not the knowledge that is valuable, It’s the practice of learning new things and understanding how things work. These are processes that you are developing so  that you are able, over time, to tackle more interesting problems, more challenging problems—problems that require many people instead of one person, and many skills instead of one.

N) If teachers keep it form-free and student-led, it can still be tied to a curriculum and an educational plan. I think a maker space is more like a like a library in that there are multiple subjects and multiple things that you can learn. What seems to be missing in school is how these subjects integrate, how they fit together in any meaningful way. Rather than saying, ‘This is science, over here is history,’ I see schools taking this idea of projects and looking at: How do they support children in higher level learning?

O) I feel like this is a shift away form a subject matter-based curriculum to a more experiential curriculum or learning. It’s still in its early stages, but I think it’s shifting around not what kids learn but how they learn.

11. A maker space is where people make things according to their personal interests.

12. The teachers’ role is enhanced in a maker space as they have to monitor and facilitate during the process.

13. Coming up with an idea of one’s own or improving one from others is key to the concept of making.

14. Contrary to structured learning, learning by doing is highly individualized.

15. America is a nation known for the idea of making things by oneself.

16. Making will be boring unless students are able to take charge.

17. Making can be related to a project, but it is created and carried out by students themselves.

18. The author suggests incorporating the idea of a maker space into a school curriculum.

19. The maker concept is a modern version of some ancient philosophical ideas.

20. Making is not taken seriously in school when students are asked to make something meaningless to them based on textbooks.

**Section C (1x10 points)**

**Directions:** There are 2 passages in this section. Each passage is followed by some questions or unfinished statements. For each of them there are four choices marked A), B), C) and D). You should decide on the best choice and mark the corresponding letter on your Answer Sheet with a single line through the centre.

**Passage One**

Questions 21 to 25 are based on the following passage.

Most kids grow up learning they cannot draw on the walls. But it might be time to unlearn that training—this summer, group of culture addicts, artists and community organizers are inviting New Yorkers to write all over the walls of an old house on Governor’s Island.

The project is called Writing On It All, and it’s a participatory writing project and artistic experiment that has happened on Governor’s Island every summer since 2013.

“Most of the participants are people who are just walking by or are on the island for other reasons, or they just kind of happen to be there,” Alexandra Chasin, artistic director of Writing On It All,tells Smithsonian.com.

The 2016 season runs through June 26 and features sessions facilitated by everyone from dancers to domestic workers. Each session has a theme, and participants are given a variety of materials and prompts and asked to cover surfaces with their thoughts and art. This year, the programs range from one that turns the house into a collaborative essay to one that explores the meaning of exile.

Governor’s Island is a national historic landmark district long used for military purposes. Now known as “New York’s shared space for art and play,” the island, which lies between Manhattan and Brooklyn in Upper New York Bay, is closed to cars but open to summer tourists who flck for festivals, picnics, adventures, as well as these “legal graffiti” Sessions.

The notes and art scribbled on the walls are an experiment in self-expression. So far, participants have ranged in age from 2 to 85. Though Chasin says the focus of the work is on the activity of writing, rather than the text that ends up getting written, some of the work that comes out of the sessions has stuck with her.

“One of the sessions that moved me the most was state violence on black women and black girls,” says Chasin, explaining that in one room, people wrote down the names of those killed because of it. “People do beautiful work and leave beautiful messages.”

**21.What does the project Writing On It All invite people to do?**

A) Unlearn their training in drawing.

B) Participate in a state graffiti show.

C) Cover the walls of an old house with graffiti.

D) Exhibit their artistic creations in an old house.

**22.What do we learn about the participants in the project?**

A) They are just culture addicts.

B) They are graffiti enthusiasts.

C) They are writers and artists.

D) They are mostly passers-by.

**23.What did the project participants do during the 2016 season?**

A) They were free to scribble on the walls whatever came to their mind.

B) They expressed their thoughts in graffiti on the theme of each session.

C) They learned the techniques of collaborative writing.

D) They were required to cooperate with other creators.

**24.What kind of place is Governor’s Island?**

A) It is a historic site that attracts tourists and artists.

B) It is an area now accessible only to tourist vehicles.

C) It is a place in Upper New York Bay formerly used for exiles.

D) It is an open area for tourists to enjoy themselves year round.

**25.What does Chasin say about the project?**

A) It just focused on the sufferings of black females.

B) It helped expand the influence of graffiti art.

C) It has started the career of many creative artists.

D) It has created some meaningful artistic works.

**Passage Two**

**Questions 26 to 30 are based on the following passage.**

Online programs to fight depression are already commercially available. While they sound efficient and cost-saving, a recent study reports that they are not effective, primarily because depressed patients are not likely to engage with them or stick with them.

The study looked at computer-assisted cognitive behavioral therapy(CBT) and found that it was no more effective in treating depression than the usual care patients receive from a primary care doctor.

Traditional CBT is considered an effective form of talk therapy for depression, helping people challenge negative thoughts and change the way they think in order to change their mood and behaviors. However, online CBT programs have been gaining popularity, with the attraction of providing low-cost help wherever someone has access to a computer.

A team of researchers from the University of Y ork conducted a randomized control trial with 691 depressed patients from 83 physician practices across England. The patients were split into three groups: one group received only usual care from a physician while the other two groups received usual care from a physician plus one of two computerized CBT programs. Participants were balanced across the three groups for age, sex, educational background, severity and duration of depression, and use of antidepressants.

After four months, the patients using the computerized CBT programs had no improvement in depression levels over the patients who were only getting usual care from their doctors.

“It’s an important, cautionary note that we shouldn’t get too carried away with the idea that a computer system can replace doctors and therapists, ” says Christopher Dowrick, a professor of primary medical care at the University of Liverpool. “We do still need the human touch or the human interaction, particularly when people are depressed.”

Being depressed can mean feeling “lost in your own small, negative, dark world,” Dowrick says. Having a person, instead of a computer, reach out to you is particularly important in combating that sense of isolation. “When you’re emotionally vulnerable, you’re even more in need of a caring human being,” he says.

**26. What does the recent study say about online CBT programs?**

A) Patients may not be able to carry them through for effective cure.

B) Patients cannot engage with them without the use of a computer.

C) They can save patients trouble visiting physicians.

D) They have been well received by a lot of patients.

**27.What has made online CBT programs increasingly popular?**

A) Their effectiveness in combating depression.

B) The low efficiency of traditional talk therapy.

C) Their easy and inexpensive access by patients.

D) The recommendation by primary care doctors.

**28. What is the major finding by researchers at the University of York?**

A) Online CBT programs are no more effective than regular care from physicians.

B) The process of treating depression is often more complicated than anticipated.

C) The combination of traditional CBT and computerized CBT is most effective.

D) Depression is a mental condition which is to be treated with extreme caution.

**29. What is Professor Dowrick’s advice concerning online CBT programs?**

A) They should not be neglected in primary care.

B) Their effectiveness should not be overestimated.

C) They should be used by strictly following instructions.

D) Their use should be encouraged by doctors and therapists.

**30. What is more important to an emotionally vulnerable person?**

A) A positive state of mind.

B) Appropriate medication.

C) Timely encouragement.

D) Human interaction.
