# Supplementary material for: Enhancing English reading motivation and performance via the ARCS model: an empirical study using the ARCS motivation scale
Source: Front Psychol. 2025 Oct 28;16:1499957. doi: 10.3389/fpsyg.2025.1499957 (PMC12602433; doi:10.3389/fpsyg.2025.1499957)
Supplement: Supplementary file 6 [file Table_1.doc]

**Confirmatory factor analysis of English reading motivation scale**


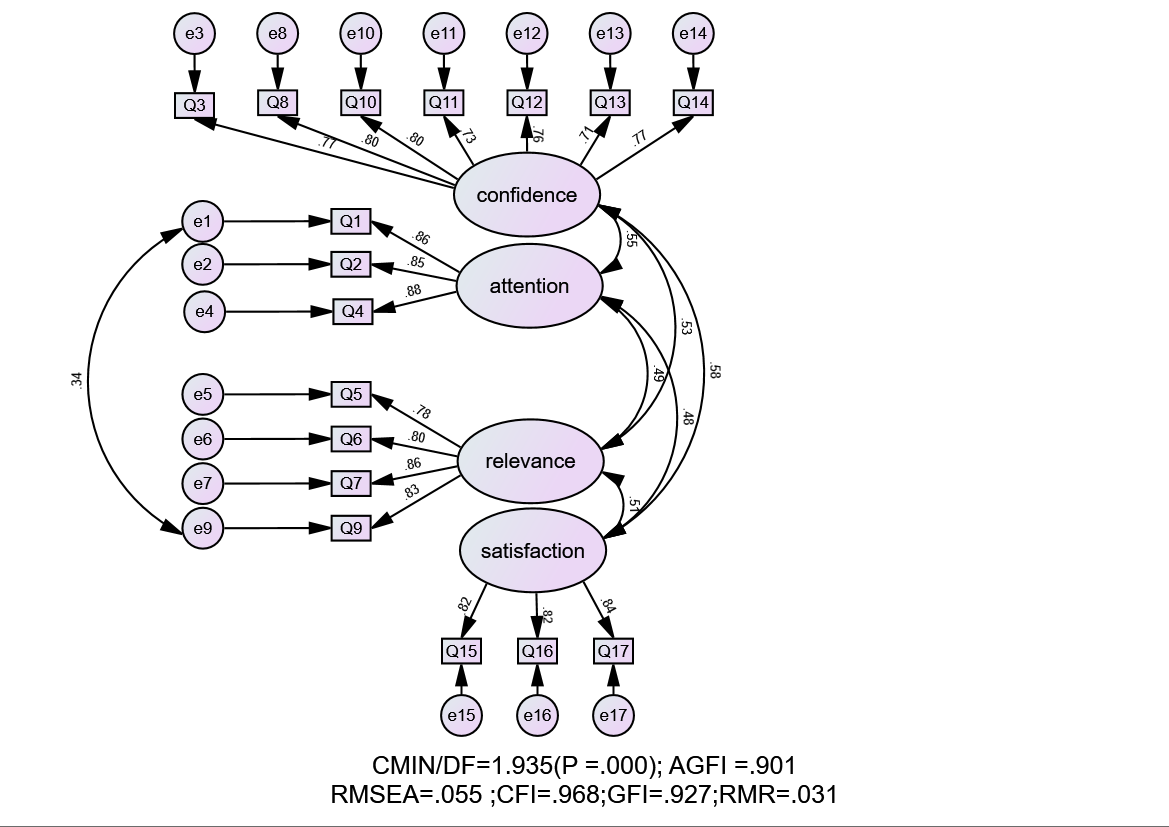


| **Estimates (Group number 1 - Default model)** | | | |  |  |  |  |  |
| --- | --- | --- | --- | --- | --- | --- | --- | --- |
| **Scalar Estimates (Group number 1 - Default model)** | | | | |  |  |  |  |
| **Maximum Likelihood Estimates** | | |  |  |  |  |  |  |
| **Regression Weights: (Group number 1 - Default model)** | | | | |  |  |  |  |
|  |  |  |  |  |  |  |  |  |
|  |  |  | **Estimate** | **S.E.** | **C.R.** | **P** | **Label** |  |
| Q3 | <--- | confidence | 1 |  |  |  |  |  |
| Q8 | <--- | confidence | 1.057 | 0.072 | 14.776 | *** |  |  |
| Q10 | <--- | confidence | 1.056 | 0.071 | 14.886 | *** |  |  |
| Q11 | <--- | confidence | 0.901 | 0.067 | 13.363 | *** |  |  |
| Q12 | <--- | confidence | 0.954 | 0.068 | 14.005 | *** |  |  |
| Q13 | <--- | confidence | 0.93 | 0.072 | 12.987 | *** |  |  |
| Q4 | <--- | attention | 0.367 | 0.05 | 7.277 | *** |  |  |
| Q2 | <--- | attention | 0.347 | 0.048 | 7.21 | *** |  |  |
| Q1 | <--- | attention | 0.362 | 0.05 | 7.234 | *** |  |  |
| Q9 | <--- | relevance | 0.341 | 0.058 | 5.915 | *** |  |  |
| Q7 | <--- | relevance | 0.377 | 0.063 | 5.944 | *** |  |  |
| Q6 | <--- | relevance | 0.335 | 0.057 | 5.858 | *** |  |  |
| Q5 | <--- | relevance | 0.329 | 0.056 | 5.834 | *** |  |  |
| Q17 | <--- | satisfaction | 0.84 | 0.114 | 7.388 | *** |  |  |
| Q16 | <--- | satisfaction | 0.893 | 0.121 | 7.35 | *** |  |  |
| Q15 | <--- | satisfaction | 0.916 | 0.125 | 7.332 | *** |  |  |
| Q14 | <--- | confidence | 1.014 | 0.071 | 14.276 | *** |  |  |
|  |  |  |  |  |  |  |  |  |
| **Standardized Regression Weights: (Group number 1 - Default model)** | | | | | |  |  |  |
|  |  |  |  |  |  |  |  |  |
|  |  |  | **Estimate** |  |  |  |  |  |
| Q3 | <--- | confidence | 0.771 |  |  |  |  |  |
| Q8 | <--- | confidence | 0.797 |  |  |  |  |  |
| Q10 | <--- | confidence | 0.802 |  |  |  |  |  |
| Q11 | <--- | confidence | 0.732 |  |  |  |  |  |
| Q12 | <--- | confidence | 0.761 |  |  |  |  |  |
| Q13 | <--- | confidence | 0.714 |  |  |  |  |  |
| Q4 | <--- | attention | 0.881 |  |  |  |  |  |
| Q2 | <--- | attention | 0.85 |  |  |  |  |  |
| Q1 | <--- | attention | 0.857 |  |  |  |  |  |
| Q9 | <--- | relevance | 0.83 |  |  |  |  |  |
| Q7 | <--- | relevance | 0.861 |  |  |  |  |  |
| Q6 | <--- | relevance | 0.795 |  |  |  |  |  |
| Q5 | <--- | relevance | 0.779 |  |  |  |  |  |
| Q17 | <--- | satisfaction | 0.838 |  |  |  |  |  |
| Q16 | <--- | satisfaction | 0.824 |  |  |  |  |  |
| Q15 | <--- | satisfaction | 0.817 |  |  |  |  |  |
| Q14 | <--- | confidence | 0.774 |  |  |  |  |  |
|  |  |  |  |  |  |  |  |  |
| **Covariances: (Group number 1 - Default model)** | | | | |  |  |  |  |
|  |  |  |  |  |  |  |  |  |
|  |  |  | **Estimate** | **S.E.** | **C.R.** | **P** | **Label** |  |
| confidence | <--> | relevance | 0.915 | 0.117 | 7.804 | *** |  |  |
| confidence | <--> | satisfaction | 0.364 | 0.068 | 5.349 | *** |  |  |
| attention | <--> | relevance | 2.753 | 0.631 | 4.361 | *** |  |  |
| attention | <--> | satisfaction | 1 |  |  |  |  |  |
| confidence | <--> | attention | 1 |  |  |  |  |  |
| relevance | <--> | satisfaction | 1 |  |  |  |  |  |
| e1 | <--> | e9 | 0.095 | 0.022 | 4.327 | *** |  |  |
|  |  |  |  |  |  |  |  |  |
| **Correlations: (Group number 1 - Default model)** | | | | |  |  |  |  |
|  |  |  |  |  |  |  |  |  |
|  |  |  | **Estimate** |  |  |  |  |  |
| confidence | <--> | relevance | 0.532 |  |  |  |  |  |
| confidence | <--> | satisfaction | 0.577 |  |  |  |  |  |
| attention | <--> | relevance | 0.49 |  |  |  |  |  |
| attention | <--> | satisfaction | 0.485 |  |  |  |  |  |
| confidence | <--> | attention | 0.555 |  |  |  |  |  |
| relevance | <--> | satisfaction | 0.508 |  |  |  |  |  |
| e1 | <--> | e9 | 0.338 |  |  |  |  |  |
|  |  |  |  |  |  |  |  |  |
| **Variances: (Group number 1 - Default model)** | | | |  |  |  |  |  |
|  |  |  |  |  |  |  |  |  |
|  |  |  | **Estimate** | **S.E.** | **C.R.** | **P** | **Label** |  |
| **confidence** |  |  | 0.551 | 0.07 | 7.844 | *** |  |  |
| **attention** |  |  | 5.895 | 1.342 | 4.391 | *** |  |  |
| **relevance** |  |  | 5.362 | 1.607 | 3.336 | *** |  |  |
| **satisfaction** |  |  | 0.722 | 0.177 | 4.07 | *** |  |  |
| **e3** |  |  | 0.375 | 0.035 | 10.744 | *** |  |  |
| **e8** |  |  | 0.354 | 0.034 | 10.431 | *** |  |  |
| **e10** |  |  | 0.342 | 0.033 | 10.36 | *** |  |  |
| **e11** |  |  | 0.388 | 0.035 | 11.116 | *** |  |  |
| **e12** |  |  | 0.364 | 0.034 | 10.849 | *** |  |  |
| **e13** |  |  | 0.459 | 0.041 | 11.247 | *** |  |  |
| **e4** |  |  | 0.228 | 0.03 | 7.547 | *** |  |  |
| **e2** |  |  | 0.274 | 0.031 | 8.807 | *** |  |  |
| **e1** |  |  | 0.28 | 0.033 | 8.491 | *** |  |  |
| **e9** |  |  | 0.281 | 0.03 | 9.213 | *** |  |  |
| **e7** |  |  | 0.266 | 0.032 | 8.305 | *** |  |  |
| **e6** |  |  | 0.35 | 0.035 | 9.989 | *** |  |  |
| **e5** |  |  | 0.378 | 0.037 | 10.256 | *** |  |  |
| **e17** |  |  | 0.215 | 0.027 | 8.014 | *** |  |  |
| **e16** |  |  | 0.272 | 0.032 | 8.475 | *** |  |  |
| **e15** |  |  | 0.301 | 0.035 | 8.684 | *** |  |  |
| **e14** |  |  | 0.38 | 0.035 | 10.717 | *** |  |  |

| **Model Fit Summary** | |  |  |  |  |
| --- | --- | --- | --- | --- | --- |
|  |  |  |  |  |  |
| **CMIN** |  |  |  |  |  |
|  |  |  |  |  |  |
| **Model** | **NPAR** | **CMIN** | **DF** | **P** | **CMIN/DF** |
| **Default model** | 41 | 216.768 | 112 | 0 | 1.935 |
| **Saturated model** | 153 | 0 | 0 |  |  |
| **Independence model** | 17 | 3429.699 | 136 | 0 | 25.218 |
|  |  |  |  |  |  |
| **RMR, GFI** |  |  |  |  |  |
|  |  |  |  |  |  |
| **Model** | **RMR** | **GFI** | **AGFI** | **PGFI** |  |
| **Default model** | 0.031 | 0.927 | 0.901 | 0.679 |  |
| **Saturated model** | 0 | 1 |  |  |  |
| **Independence model** | 0.381 | 0.249 | 0.155 | 0.221 |  |
|  |  |  |  |  |  |
| **Baseline Comparisons** | |  |  |  |  |
|  |  |  |  |  |  |
| **Model** | **NFI** | **RFI** | **IFI** | **TLI** | **CFI** |
|  | **Delta1** | **rho1** | **Delta2** | **rho2** |  |
| **Default model** | 0.937 | 0.923 | 0.968 | 0.961 | 0.968 |
| **Saturated model** | 1 |  | 1 |  | 1 |
| **Independence model** | 0 | 0 | 0 | 0 | 0 |
|  |  |  |  |  |  |
| **Parsimony-Adjusted Measures** | | |  |  |  |
|  |  |  |  |  |  |
| **Model** | **PRATIO** | **PNFI** | **PCFI** |  |  |
| **Default model** | 0.824 | 0.771 | 0.797 |  |  |
| **Saturated model** | 0 | 0 | 0 |  |  |
| **Independence model** | 1 | 0 | 0 |  |  |
|  |  |  |  |  |  |
| **NCP** |  |  |  |  |  |
|  |  |  |  |  |  |
| **Model** | **NCP** | **LO 90** | **HI 90** |  |  |
| **Default model** | 104.768 | 66.904 | 150.432 |  |  |
| **Saturated model** | 0 | 0 | 0 |  |  |
| **Independence model** | 3293.699 | 3106.548 | 3488.16 |  |  |
|  |  |  |  |  |  |
| **FMIN** |  |  |  |  |  |
|  |  |  |  |  |  |
| **Model** | **FMIN** | **F0** | **LO 90** | **HI 90** |  |
| **Default model** | 0.699 | 0.338 | 0.216 | 0.485 |  |
| **Saturated model** | 0 | 0 | 0 | 0 |  |
| **Independence model** | 11.064 | 10.625 | 10.021 | 11.252 |  |
|  |  |  |  |  |  |
| **RMSEA** |  |  |  |  |  |
|  |  |  |  |  |  |
| **Model** | **RMSEA** | **LO 90** | **HI 90** | **PCLOSE** |  |
| **Default model** | 0.055 | 0.044 | 0.066 | 0.221 |  |
| **Independence model** | 0.28 | 0.271 | 0.288 | 0 |  |
|  |  |  |  |  |  |
| **AIC** |  |  |  |  |  |
|  |  |  |  |  |  |
| **Model** | **AIC** | **BCC** | **BIC** | **CAIC** |  |
| **Default model** | 298.768 | 303.822 | 452.099 | 493.099 |  |
| **Saturated model** | 306 | 324.863 | 878.188 | 1031.188 |  |
| **Independence model** | 3463.699 | 3465.795 | 3527.276 | 3544.276 |  |
|  |  |  |  |  |  |
| **ECVI** |  |  |  |  |  |
|  |  |  |  |  |  |
| **Model** | **ECVI** | **LO 90** | **HI 90** | **MECVI** |  |
| **Default model** | 0.964 | 0.842 | 1.111 | 0.98 |  |
| **Saturated model** | 0.987 | 0.987 | 0.987 | 1.048 |  |
| **Independence model** | 11.173 | 10.57 | 11.801 | 11.18 |  |
|  |  |  |  |  |  |
| **HOELTER** | |  |  |  |  |
|  |  |  |  |  |  |
| **Model** | **HOELTER** | **HOELTER** |  |  |  |
|  | **0.05** | **0.01** |  |  |  |
| **Default model** | 197 | 215 |  |  |  |
| **Independence model** | 15 | 17 |  |  |  |
