# Supplementary material for: Enhancing English reading motivation and performance via the ARCS model: an empirical study using the ARCS motivation scale
Source: Front Psychol. 2025 Oct 28;16:1499957. doi: 10.3389/fpsyg.2025.1499957 (PMC12602433; doi:10.3389/fpsyg.2025.1499957)
Supplement: Supplementary file 7 [file Table_2.doc]

**Confirmatory Factor Analysis of the Model--Confidence**


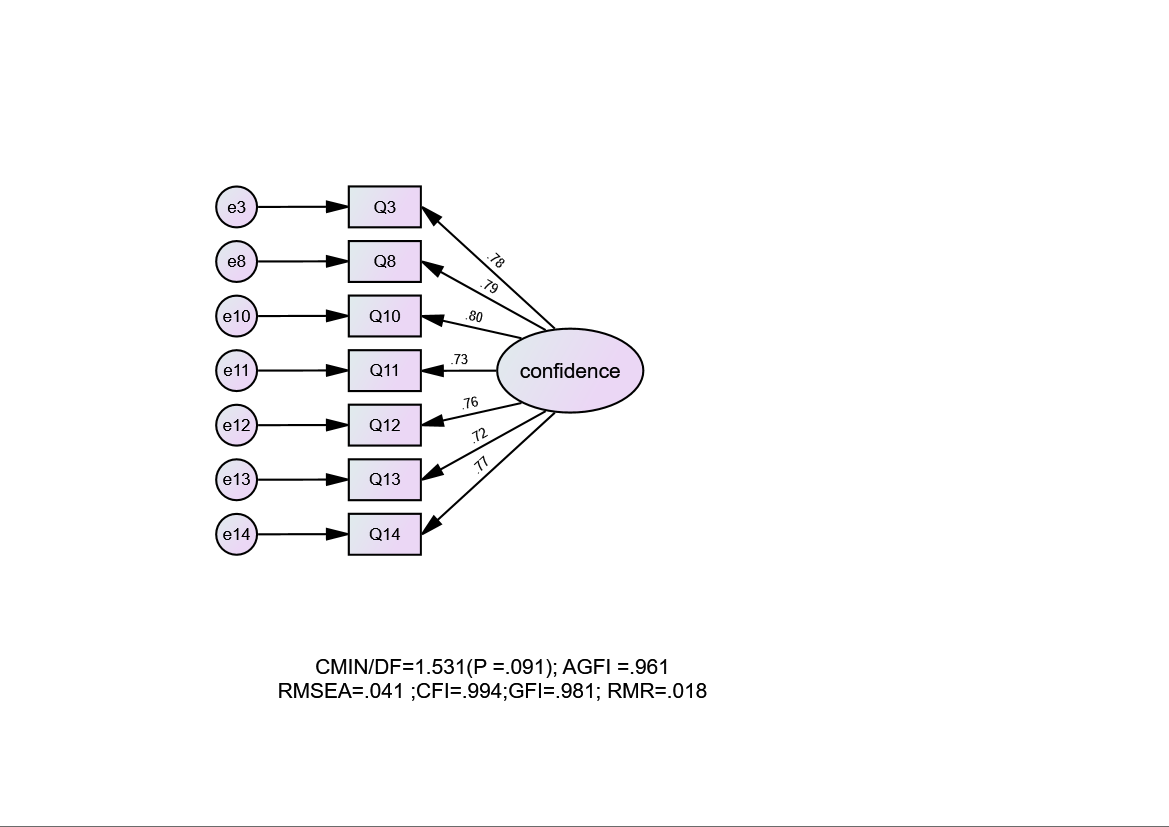


| **Estimates (Group number 1 - Default model)** | | | |  |  |  |  |
| --- | --- | --- | --- | --- | --- | --- | --- |
| **Scalar Estimates (Group number 1 - Default model)** | | | | |  |  |  |
| **Maximum Likelihood Estimates** | | |  |  |  |  |  |
| **Regression Weights: (Group number 1 - Default model)** | | | | |  |  |  |
|  |  |  | **Estimate** | **S.E.** | **C.R.** | **P** | **Label** |
| Q14 | <--- | confidence | 1 |  |  |  |  |
| Q13 | <--- | confidence | 0.924 | 0.071 | 13.1 | *** |  |
| Q12 | <--- | confidence | 0.938 | 0.067 | 13.942 | *** |  |
| Q11 | <--- | confidence | 0.885 | 0.067 | 13.294 | *** |  |
| Q10 | <--- | confidence | 1.04 | 0.07 | 14.851 | *** |  |
| Q8 | <--- | confidence | 1.034 | 0.071 | 14.627 | *** |  |
| Q3 | <--- | confidence | 0.992 | 0.069 | 14.309 | *** |  |
| **Standardized Regression Weights: (Group number 1 - Default model)** | | | | | |  |  |
|  |  |  | **Estimate** |  |  |  |  |
| Q14 | <--- | confidence | 0.775 |  |  |  |  |
| Q13 | <--- | confidence | 0.72 |  |  |  |  |
| Q12 | <--- | confidence | 0.76 |  |  |  |  |
| Q11 | <--- | confidence | 0.729 |  |  |  |  |
| Q10 | <--- | confidence | 0.801 |  |  |  |  |
| Q8 | <--- | confidence | 0.791 |  |  |  |  |
| Q3 | <--- | confidence | 0.776 |  |  |  |  |
| **Variances: (Group number 1 - Default model)** | | | |  |  |  |  |
|  |  |  | **Estimate** | **S.E.** | **C.R.** | **P** | **Label** |
| **confidence** |  |  | 0.568 | 0.072 | 7.864 | *** |  |
| **e14** |  |  | 0.379 | 0.036 | 10.569 | *** |  |
| **e13** |  |  | 0.45 | 0.041 | 11.103 | *** |  |
| **e12** |  |  | 0.366 | 0.034 | 10.742 | *** |  |
| **e11** |  |  | 0.391 | 0.035 | 11.028 | *** |  |
| **e10** |  |  | 0.343 | 0.034 | 10.198 | *** |  |
| **e8** |  |  | 0.363 | 0.035 | 10.352 | *** |  |
| **e3** |  |  | 0.368 | 0.035 | 10.546 | *** |  |

| **Model Fit Summary** | |  |  |  |  |  |
| --- | --- | --- | --- | --- | --- | --- |
| **CMIN** |  |  |  |  |  |  |
| **Model** | **NPAR** | **CMIN** | **DF** | **P** | **CMIN/DF** |  |
| **Default model** | 14 | 21.427 | 14 | 0.091 | 1.531 |  |
| **Saturated model** | 28 | 0 | 0 |  |  |  |
| **Independence model** | 7 | 1198.26 | 21 | 0 | 57.06 |  |
| **RMR, GFI** |  |  |  |  |  |  |
| **Model** | **RMR** | **GFI** | **AGFI** | **PGFI** |  |  |
| **Default model** | 0.018 | 0.981 | 0.961 | 0.49 |  |  |
| **Saturated model** | 0 | 1 |  |  |  |  |
| **Independence model** | 0.467 | 0.327 | 0.103 | 0.245 |  |  |
| **Baseline Comparisons** | |  |  |  |  |  |
| **Model** | **NFI** | **RFI** | **IFI** | **TLI** | **CFI** |  |
|  | **Delta1** | **rho1** | **Delta2** | **rho2** |  |  |
| **Default model** | 0.982 | 0.973 | 0.994 | 0.991 | 0.994 |  |
| **Saturated model** | 1 |  | 1 |  | 1 |  |
| **Independence model** | 0 | 0 | 0 | 0 | 0 |  |
| **Parsimony-Adjusted Measures** | | |  |  |  |  |
| **Model** | **PRATIO** | **PNFI** | **PCFI** |  |  |  |
| **Default model** | 0.667 | 0.655 | 0.662 |  |  |  |
| **Saturated model** | 0 | 0 | 0 |  |  |  |
| **Independence model** | 1 | 0 | 0 |  |  |  |
| **NCP** |  |  |  |  |  |  |
| **Model** | **NCP** | **LO 90** | **HI 90** |  |  |  |
| **Default model** | 7.427 | 0 | 24.043 |  |  |  |
| **Saturated model** | 0 | 0 | 0 |  |  |  |
| **Independence model** | 1177.26 | 1067.536 | 1294.367 |  |  |  |
| **FMIN** |  |  |  |  |  |  |
| **Model** | **FMIN** | **F0** | **LO 90** | **HI 90** |  |  |
| **Default model** | 0.069 | 0.024 | 0 | 0.078 |  |  |
| **Saturated model** | 0 | 0 | 0 | 0 |  |  |
| **Independence model** | 3.865 | 3.798 | 3.444 | 4.175 |  |  |
| **RMSEA** |  |  |  |  |  |  |
| **Model** | **RMSEA** | **LO 90** | **HI 90** | **PCLOSE** |  |  |
| **Default model** | 0.041 | 0 | 0.074 | 0.624 |  |  |
| **Independence model** | 0.425 | 0.405 | 0.446 | 0 |  |  |
| **AIC** |  |  |  |  |  |  |
| **Model** | **AIC** | **BCC** | **BIC** | **CAIC** |  |  |
| **Default model** | 49.427 | 50.169 | 101.784 | 115.784 |  |  |
| **Saturated model** | 56 | 57.483 | 160.714 | 188.714 |  |  |
| **Independence model** | 1212.26 | 1212.63 | 1238.438 | 1245.438 |  |  |
| **ECVI** |  |  |  |  |  |  |
| **Model** | **ECVI** | **LO 90** | **HI 90** | **MECVI** |  |  |
| **Default model** | 0.159 | 0.135 | 0.213 | 0.162 |  |  |
| **Saturated model** | 0.181 | 0.181 | 0.181 | 0.185 |  |  |
| **Independence model** | 3.911 | 3.557 | 4.288 | 3.912 |  |  |
| **HOELTER** | |  |  |  |  |  |
| **Model** | **HOELTER** | **HOELTER** |  |  |  |  |
|  | **0.05** | **0.01** |  |  |  |  |
| **Default model** | 343 | 422 |  |  |  |  |
| **Independence model** | 9 | 11 |  |  |  |  |
