# Supplementary material for: Enhancing English reading motivation and performance via the ARCS model: an empirical study using the ARCS motivation scale
Source: Front Psychol. 2025 Oct 28;16:1499957. doi: 10.3389/fpsyg.2025.1499957 (PMC12602433; doi:10.3389/fpsyg.2025.1499957)
Supplement: Supplementary file 8 [file Table_3.doc]

**Confirmatory Factor Analysis of the Model--Relevance**


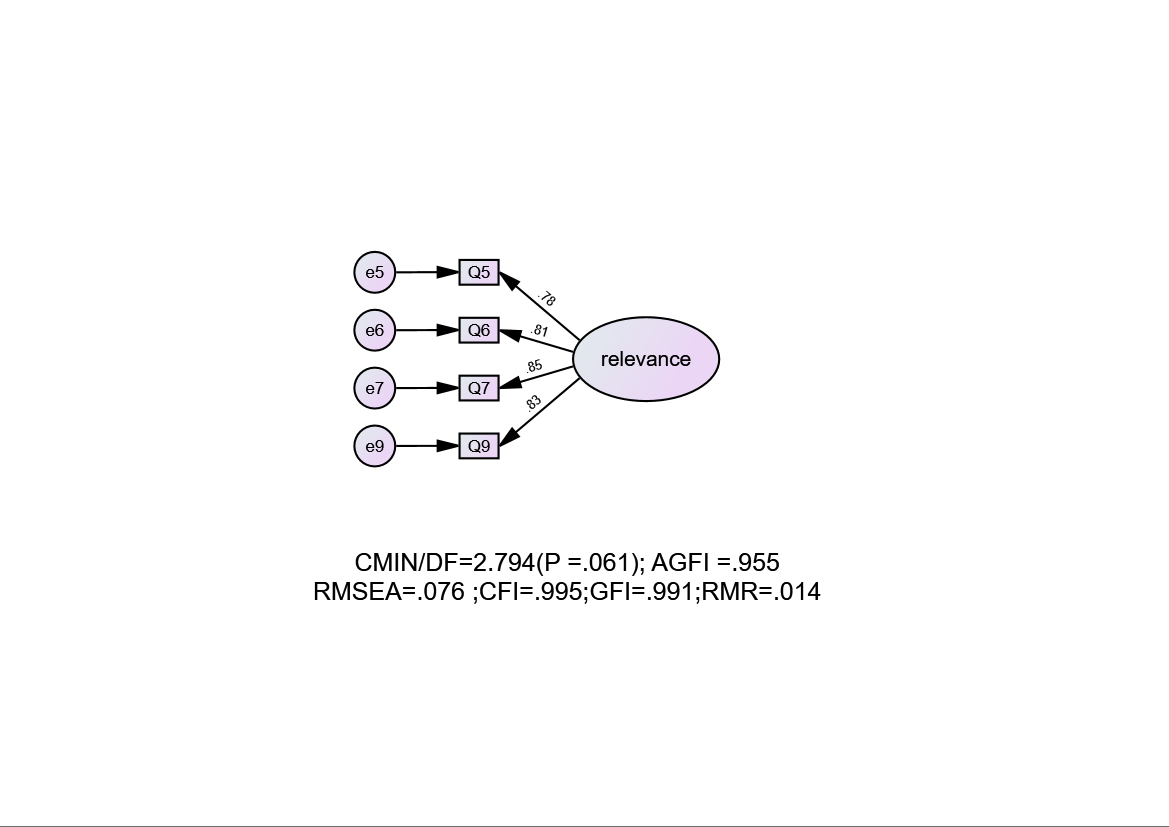


**Estimates**

| **Estimates (Group number 1 - Default model)** | | | | | |  | | | | | | |
| --- | --- | --- | --- | --- | --- | --- | --- | --- | --- | --- | --- | --- |
| **Scalar Estimates (Group number 1 - Default model)** | | | |  |  | | | |  | |  | |
| **Maximum Likelihood Estimates** | | |  | | |  | |  | |  | |  |
| **Regression Weights: (Group number 1 - Default model)** | | | |  |  | | | |  | |  | |
|  |  |  | **Estimate** | | | **S.E.** | | **C.R.** | | **P** | | **Label** |
| Q9 | <--- | relevance | 1.032 | | | 0.068 | | 15.176 | | *** | |  |
| Q7 | <--- | relevance | 1.136 | | | 0.073 | | 15.605 | | *** | |  |
| Q6 | <--- | relevance | 1.036 | | | 0.07 | | 14.748 | | *** | |  |
| Q5 | <--- | relevance | 1 | | |  | |  | |  | |  |
|  |  |  |  | | |  | |  | |  | |  |
| **Standardized Regression Weights: (Group number 1 - Default model)** | | | | | | |  | |  | |  | |
|  |  |  | **Estimate** | | |  | |  | |  | |  |
| Q9 | <--- | relevance | 0.83 | | |  | |  | |  | |  |
| Q7 | <--- | relevance | 0.853 | | |  | |  | |  | |  |
| Q6 | <--- | relevance | 0.808 | | |  | |  | |  | |  |
| Q5 | <--- | relevance | 0.778 | | |  | |  | |  | |  |
| **Variances: (Group number 1 - Default model)** | | | | | |  | | | | | | |
|  |  |  |  | | |  | |  | |  | |  |
|  |  |  | **Estimate** | | | **S.E.** | | **C.R.** | | **P** | | **Label** |
| **relevance** |  |  | 0.58 | | | 0.074 | | 7.823 | | *** | |  |
| **e9** |  |  | 0.28 | | | 0.031 | | 8.948 | | *** | |  |
| **e7** |  |  | 0.281 | | | 0.034 | | 8.225 | | *** | |  |
| **e6** |  |  | 0.33 | | | 0.035 | | 9.48 | | *** | |  |
| **e5** |  |  | 0.379 | | | 0.038 | | 10.06 | | *** | |  |

| **Model Fit Summary** | |  | | | | |
| --- | --- | --- | --- | --- | --- | --- |
|  | |  | | | | |
| **CMIN** |  |  |  |  |  |  |
| **Model** | **NPAR** | **CMIN** | **DF** | **P** | **CMIN/DF** |  |
| **Default model** | 8 | 5.589 | 2 | 0.061 | 2.794 |  |
| **Saturated model** | 10 | 0 | 0 |  |  |  |
| **Independence model** | 4 | 695.17 | 6 | 0 | 115.862 |  |
| **RMR, GFI** |  |  |  |  |  |  |
| **Model** | **RMR** | **GFI** | **AGFI** | **PGFI** |  |  |
| **Default model** | 0.014 | 0.991 | 0.955 | 0.198 |  |  |
| **Saturated model** | 0 | 1 |  |  |  |  |
| **Independence model** | 0.497 | 0.427 | 0.045 | 0.256 |  |  |
| **Baseline Comparisons** | |  |  |  |  |  |
| **Model** | **NFI** | **RFI** | **IFI** | **TLI** | **CFI** |  |
|  | **Delta1** | **rho1** | **Delta2** | **rho2** |  |  |
| **Default model** | 0.992 | 0.976 | 0.995 | 0.984 | 0.995 |  |
| **Saturated model** | 1 |  | 1 |  | 1 |  |
| **Independence model** | 0 | 0 | 0 | 0 | 0 |  |
| **Parsimony-Adjusted Measures** | | |  |  |  |  |
| **Model** | **PRATIO** | **PNFI** | **PCFI** |  |  |  |
| **Default model** | 0.333 | 0.331 | 0.332 |  |  |  |
| **Saturated model** | 0 | 0 | 0 |  |  |  |
| **Independence model** | 1 | 0 | 0 |  |  |  |
| **NCP** |  |  |  |  |  |  |
| **Model** | **NCP** | **LO 90** | **HI 90** |  |  |  |
| **Default model** | 3.589 | 0 | 14.755 |  |  |  |
| **Saturated model** | 0 | 0 | 0 |  |  |  |
| **Independence model** | 689.17 | 606.279 | 779.456 |  |  |  |
| **FMIN** |  |  |  |  |  |  |
| **Model** | **FMIN** | **F0** | **LO 90** | **HI 90** |  |  |
| **Default model** | 0.018 | 0.012 | 0 | 0.048 |  |  |
| **Saturated model** | 0 | 0 | 0 | 0 |  |  |
| **Independence model** | 2.242 | 2.223 | 1.956 | 2.514 |  |  |
| **RMSEA** |  |  |  |  |  |  |
| **Model** | **RMSEA** | **LO 90** | **HI 90** | **PCLOSE** |  |  |
| **Default model** | 0.076 | 0 | 0.154 | 0.208 |  |  |
| **Independence model** | 0.609 | 0.571 | 0.647 | 0 |  |  |
| **AIC** |  |  |  |  |  |  |
| **Model** | **AIC** | **BCC** | **BIC** | **CAIC** |  |  |
| **Default model** | 21.589 | 21.851 | 51.507 | 59.507 |  |  |
| **Saturated model** | 20 | 20.328 | 57.398 | 67.398 |  |  |
| **Independence model** | 703.17 | 703.301 | 718.129 | 722.129 |  |  |
| **ECVI** |  |  |  |  |  |  |
| **Model** | **ECVI** | **LO 90** | **HI 90** | **MECVI** |  |  |
| **Default model** | 0.07 | 0.058 | 0.106 | 0.07 |  |  |
| **Saturated model** | 0.065 | 0.065 | 0.065 | 0.066 |  |  |
| **Independence model** | 2.268 | 2.001 | 2.56 | 2.269 |  |  |
| **HOELTER** | |  |  |  |  |  |
| **Model** | **HOELTER** | **HOELTER** |  |  |  |  |
|  | **0.05** | **0.01** |  |  |  |  |
| **Default model** | 333 | 511 |  |  |  |  |
| **Independence model** | 6 | 8 |  |  |  |  |
