# Supplementary material for: Enhancing English reading motivation and performance via the ARCS model: an empirical study using the ARCS motivation scale
Source: Front Psychol. 2025 Oct 28;16:1499957. doi: 10.3389/fpsyg.2025.1499957 (PMC12602433; doi:10.3389/fpsyg.2025.1499957)
Supplement: Supplementary file 9 [file Table_4.doc]

**Exploratory** Factor Analysis

| **KMO and Bartlett's Test** | | |
| --- | --- | --- |
| Kaiser-Meyer-Olkin Measure of Sampling Adequacy. | | .909 |
| Bartlett's Test of Sphericity | Approx. Chi-Square | 2655.620 |
|  | df | 136 |
|  | Sig. | 0.000 |

| **Communalities** | | |
| --- | --- | --- |
|  | Initial | Extraction |
| Q1 | 1.000 | .700 |
| Q2 | 1.000 | .799 |
| Q3 | 1.000 | .617 |
| Q4 | 1.000 | .680 |
| Q5 | 1.000 | .597 |
| Q6 | 1.000 | .744 |
| Q7 | 1.000 | .604 |
| Q8 | 1.000 | .637 |
| Q9 | 1.000 | .608 |
| Q10 | 1.000 | .602 |
| Q11 | 1.000 | .635 |
| Q12 | 1.000 | .548 |
| Q13 | 1.000 | .593 |
| Q14 | 1.000 | .497 |
| Q15 | 1.000 | .614 |
| Q16 | 1.000 | .883 |
| Q17 | 1.000 | .871 |


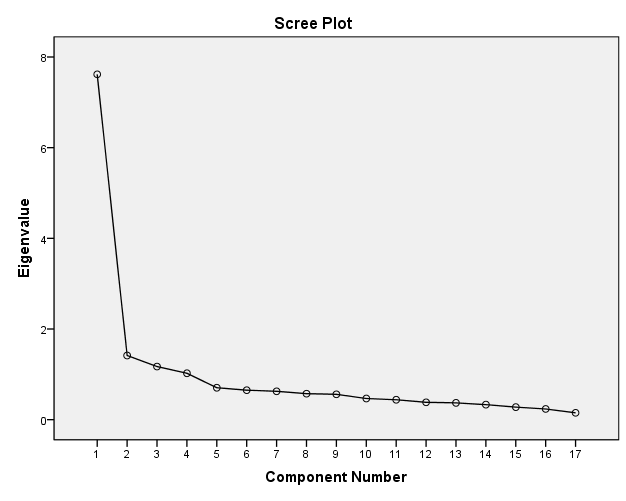


| Component | Initial Eigenvalues | | | Extraction Sums of Squared Loadings | | |
| --- | --- | --- | --- | --- | --- | --- |
|  | Total | % of Variance | Cumulative % | Total | % of Variance | Cumulative % |
| 1 | 7.618 | 44.811 | 44.811 | 7.618 | 44.811 | 44.811 |
| 2 | 1.416 | 8.329 | 53.14 | 1.416 | 8.329 | 53.14 |
| 3 | 1.172 | 6.891 | 60.031 | 1.172 | 6.891 | 60.031 |
| 4 | 1.024 | 6.022 | 66.053 | 1.024 | 6.022 | 66.053 |
| 5 | 0.705 | 4.144 | 70.198 |  |  |  |
| 6 | 0.651 | 3.827 | 74.025 |  |  |  |
| 7 | 0.626 | 3.684 | 77.709 |  |  |  |
| 8 | 0.573 | 3.373 | 81.081 |  |  |  |
| 9 | 0.559 | 3.291 | 84.373 |  |  |  |
| 10 | 0.467 | 2.748 | 87.12 |  |  |  |
| 11 | 0.439 | 2.583 | 89.703 |  |  |  |
| 12 | 0.383 | 2.255 | 91.958 |  |  |  |
| 13 | 0.371 | 2.181 | 94.139 |  |  |  |
| 14 | 0.332 | 1.954 | 96.092 |  |  |  |
| 15 | 0.277 | 1.63 | 97.722 |  |  |  |
| 16 | 0.236 | 1.388 | 99.11 |  |  |  |
| 17 | 0.151 | 0.89 | 100 |  |  |  |

Extraction Method: Principal Component Analysis.

| **Rotated Component Matrix^a^** | | | | |
| --- | --- | --- | --- | --- |
|  | Component | | | |
|  | 1 | 2 | 3 | 4 |
| Q8 | .738 |  |  |  |
| Q11 | .710 |  |  |  |
| Q10 | .696 |  |  |  |
| Q13 | .678 |  |  |  |
| Q12 | .672 |  |  |  |
| Q14 | .627 |  |  |  |
| Q3 | .540 |  | .515 |  |
| Q6 |  | .787 |  |  |
| Q5 |  | .692 |  |  |
| Q7 |  | .653 |  |  |
| Q9 |  | .594 |  |  |
| Q2 |  |  | .847 |  |
| Q1 |  |  | .748 |  |
| Q4 |  |  | .694 |  |
| Q16 |  |  |  | .873 |
| Q17 |  |  |  | .848 |
| Q15 |  |  |  | .538 |

| **Component Transformation Matrix** | | | | |
| --- | --- | --- | --- | --- |
| Component | 1 | 2 | 3 | 4 |
| 1 | .641 | .474 | .427 | .428 |
| 2 | -.579 | .325 | .718 | -.208 |
| 3 | .502 | -.279 | .313 | -.756 |
| 4 | .032 | .770 | -.452 | -.450 |

Extraction Method: Principal Component Analysis.

Rotation Method: Varimax with Kaiser Normalization.
