# Supplementary material for: Enhancing English reading motivation and performance via the ARCS model: an empirical study using the ARCS motivation scale
Source: Front Psychol. 2025 Oct 28;16:1499957. doi: 10.3389/fpsyg.2025.1499957 (PMC12602433; doi:10.3389/fpsyg.2025.1499957)
Supplement: Supplementary file 10 [file Table_5.doc]

**Reliability test of English reading motivation scale before exploratory factor analysis**

Scale: ALL VARIABLES

| **Case Processing Summary** | | | |
| --- | --- | --- | --- |
|  | | N | % |
| Cases | Valid | 300 | 100.0 |
|  | Excluded^a^ | 0 | 0.0 |
|  | Total | 300 | 100.0 |

a. Listwise deletion based on all variables in the procedure.

| **Reliability Statistics** | |
| --- | --- |
| Cronbach's Alpha | N of Items |
| .922 | 17 |

**Item-Total Statistics**

|  | Scale Mean if Item Deleted | Scale Variance if Item Deleted | Corrected Item-Total Correlation | Cronbach's Alpha if Item Deleted |
| --- | --- | --- | --- | --- |
| Q1 | 55.67 | 103.198 | .528 | .920 |
| Q2 | 55.51 | 102.492 | .538 | .920 |
| Q3 | 55.37 | 101.044 | .612 | .918 |
| Q4 | 55.46 | 101.152 | .635 | .917 |
| Q5 | 55.45 | 101.225 | .556 | .919 |
| Q6 | 55.14 | 99.439 | .629 | .917 |
| Q7 | 55.11 | 100.343 | .631 | .917 |
| Q8 | 55.38 | 100.550 | .585 | .919 |
| Q9 | 55.37 | 100.454 | .642 | .917 |
| Q10 | 55.20 | 101.231 | .616 | .918 |
| Q11 | 55.18 | 101.192 | .673 | .916 |
| Q12 | 55.31 | 102.081 | .605 | .918 |
| Q13 | 55.39 | 100.211 | .658 | .917 |
| Q14 | 55.35 | 103.104 | .573 | .919 |
| Q15 | 55.28 | 98.390 | .694 | .915 |
| Q16 | 54.96 | 101.008 | .628 | .917 |
| Q17 | 54.98 | 99.809 | .652 | .917 |

**Scale Statistics**

| **Scale Statistics** | | | |
| --- | --- | --- | --- |
| Mean | Variance | Std. Deviation | N of Items |
| 58.76 | 113.462 | 10.652 | 17 |
