# Supplementary material for: Enhancing English reading motivation and performance via the ARCS model: an empirical study using the ARCS motivation scale
Source: Front Psychol. 2025 Oct 28;16:1499957. doi: 10.3389/fpsyg.2025.1499957 (PMC12602433; doi:10.3389/fpsyg.2025.1499957)
Supplement: Supplementary file 11 [file Table_6.doc]

**Reliability test of factor 1--Attention**

| **Case Processing Summary** | | | |
| --- | --- | --- | --- |
|  | | N | % |
| Cases | Valid | 300 | 100.0 |
|  | Excluded^a^ | 0 | 0.0 |
|  | Total | 300 | 100.0 |
| a. Listwise deletion based on all variables in the procedure. | | | |

| **Reliability Statistics** | |
| --- | --- |
| Cronbach's Alpha | N of Items |
| .799 | 3 |

| **Item-Total Statistics** | | | | |
| --- | --- | --- | --- | --- |
|  | Scale Mean if Item Deleted | Scale Variance if Item Deleted | Corrected Item-Total Correlation | Cronbach's Alpha if Item Deleted |
| Q1 | 6.54 | 2.671 | .623 | .748 |
| Q2 | 6.38 | 2.303 | .740 | .618 |
| Q4 | 6.33 | 2.731 | .575 | .796 |

**Reliability test of factor 2--Relevance**

| **Case Processing Summary** | | | | | | | |  | |
| --- | --- | --- | --- | --- | --- | --- | --- | --- | --- |
|  | | | | N | | % | |  | |
| Cases | | Valid | | 300 | | 100.0 | |  | |
|  |  | Excluded^a^ | | 0 | | 0.0 | |  | |
|  |  | Total | | 300 | | 100.0 | |  | |
| a. Listwise deletion based on all variables in the procedure. | | | | | | | |  | |
|  | |  | | |  |  | |  | |
| **Reliability Statistics** | | | | |  |  | |  | |
| Cronbach's Alpha | | | N of Items | |  |  | |  | |
| .806 | | | 4 | |  |  | |  | |
|  | | |  | |  |  | |  | |
| **Item-Total Statistics** | | | | | | | | | |
|  | Scale Mean if Item Deleted | | | Scale Variance if Item Deleted | | | Corrected Item-Total Correlation | | Cronbach's Alpha if Item Deleted |
| Q5 | 10.66 | | | 6.052 | | | .564 | | .785 |
| Q6 | 10.34 | | | 5.397 | | | .705 | | .714 |
| Q7 | 10.31 | | | 5.940 | | | .632 | | .752 |
| Q9 | 10.57 | | | 6.192 | | | .590 | | .772 |

**Reliability test of factor 3--Confidence**

| **Case Processing Summary** | | | | | | | | |  | |
| --- | --- | --- | --- | --- | --- | --- | --- | --- | --- | --- |
|  | | | | N | | % | | |  | |
| Cases | | Valid | | 300 | | 100.0 | | |  | |
|  |  | Excluded^a^ | | 0 | | 0.0 | | |  | |
|  |  | Total | | 300 | | 100.0 | | |  | |
| a. Listwise deletion based on all variables in the procedure. | | | | | | | | |  | |
|  | |  | | |  | |  | |  | |
| **Reliability Statistics** | | | | |  | |  | |  | |
| Cronbach's Alpha | | | N of Items | |  | |  | |  | |
| .864 | | | 7 | |  | |  | |  | |
|  | | |  | |  | |  | |  | |
| **Item-Total Statistics** | | | | | | | | | | |
|  | Scale Mean if Item Deleted | | | Scale Variance if Item Deleted | | | | Corrected Item-Total Correlation | | Cronbach's Alpha if Item Deleted |
| Q3 | 20.74 | | | 17.056 | | | | .575 | | .853 |
| Q8 | 20.74 | | | 16.252 | | | | .626 | | .847 |
| Q10 | 20.57 | | | 16.634 | | | | .656 | | .842 |
| Q11 | 20.55 | | | 16.784 | | | | .699 | | .837 |
| Q12 | 20.68 | | | 17.089 | | | | .632 | | .846 |
| Q13 | 20.75 | | | 16.427 | | | | .667 | | .840 |
| Q14 | 20.71 | | | 17.510 | | | | .602 | | .850 |

**Reliability test of factor 4--Satisfaction**

| **Case Processing Summary** | | | | | | | | |  | |
| --- | --- | --- | --- | --- | --- | --- | --- | --- | --- | --- |
|  | | | | N | | % | | |  | |
| Cases | | Valid | | 300 | | 100.0 | | |  | |
|  |  | Excluded^a^ | | 0 | | 0.0 | | |  | |
|  |  | Total | | 300 | | 100.0 | | |  | |
| a. Listwise deletion based on all variables in the procedure. | | | | | | | | |  | |
|  | |  | | |  | |  | |  | |
| **Reliability Statistics** | | | | |  | |  | |  | |
| Cronbach's Alpha | | | N of Items | |  | |  | |  | |
| .857 | | | 3 | |  | |  | |  | |
|  | | |  | |  | |  | |  | |
| **Item-Total Statistics** | | | | | | | | | | |
|  | Scale Mean if Item Deleted | | | Scale Variance if Item Deleted | | | | Corrected Item-Total Correlation | | Cronbach's Alpha if Item Deleted |
| Q15 | 7.57 | | | 3.296 | | | | .617 | | .910 |
| Q16 | 7.25 | | | 3.171 | | | | .795 | | .744 |
| Q17 | 7.27 | | | 2.989 | | | | .794 | | .739 |
|  |  | | |  | | | |  | |  |
