# Supplementary material for: Enhancing English reading motivation and performance via the ARCS model: an empirical study using the ARCS motivation scale
Source: Front Psychol. 2025 Oct 28;16:1499957. doi: 10.3389/fpsyg.2025.1499957 (PMC12602433; doi:10.3389/fpsyg.2025.1499957)
Supplement: Supplementary file 12 [file Table_7.doc]

**1, Differences in pre-test scores of English reading motivation between the experimental class and the control class (1=experimental; 2=control)**

| **Group Statistics** | | | | | |
| --- | --- | --- | --- | --- | --- |
| group | | N | Mean | Std. Deviation | Std. Error Mean |
| Pretest­­_attention | 1 | 40 | 2.93 | 0.72 | 0.11 |
|  | 2 | 40 | 3.05 | 0.79 | 0.12 |
| Pretest_relevance | 1 | 40 | 2.64 | 0.99 | 0.16 |
|  | 2 | 40 | 2.78 | 0.77 | 0.12 |
| Pretest_confidence | 1 | 40 | 2.91 | 1.01 | 0.16 |
|  | 2 | 40 | 2.96 | 0.95 | 0.15 |
| Pretest_satisfaction | 1 | 40 | 2.86 | 0.91 | 0.14 |
|  | 2 | 40 | 2.99 | 0.99 | 0.16 |
| Pretest_total  motivation | 1 | 40 | 2.83 | 0.67 | 0.11 |
|  | 2 | 40 | 2.94 | 0.64 | 0.10 |

| **Independent Samples Test** | | | | | | | | | | |
| --- | --- | --- | --- | --- | --- | --- | --- | --- | --- | --- |
|  | | Levene's Test for Equality of Variances | | t-test for Equality of Means | | | | | | |
|  |  | F | Sig. | t | df | Sig. (2-tailed) | Mean Difference | Std. Error Difference | 95% Confidence Interval of the Difference | |
|  |  |  |  |  |  |  |  |  | Lower | Upper |
| Pretest_attention | Equal variances assumed | .022 | .882 | -.690 | 78 | .492 | -0.117 | 0.169 | -0.453 | 0.220 |
|  | Equal variances not assumed |  |  | -.690 | 77.486 | .492 | -0.117 | 0.169 | -0.453 | 0.220 |
| Pretest_relevance | Equal variances assumed | 2.785 | .099 | -.693 | 78 | .491 | -0.138 | 0.199 | -0.533 | 0.258 |
|  | Equal variances not assumed |  |  | -.693 | 73.743 | .491 | -0.138 | 0.199 | -0.533 | 0.258 |
| Pretest_confidence | Equal variances assumed | .301 | .585 | -.227 | 78 | .821 | -0.050 | 0.220 | -0.488 | 0.388 |
|  | Equal variances not assumed |  |  | -.227 | 77.709 | .821 | -0.050 | 0.220 | -0.488 | 0.388 |
| Pretest_satisfaction | Equal variances assumed | .163 | .688 | -.629 | 78 | .531 | -0.133 | 0.212 | -0.555 | 0.289 |
|  | Equal variances not assumed |  |  | -.629 | 77.489 | .531 | -0.133 | 0.212 | -0.555 | 0.289 |
| Pretest_total motivation | Equal variances assumed | .003 | .959 | -.748 | 78 | .457 | -0.109 | 0.146 | -0.400 | 0.182 |
|  | Equal variances not assumed |  |  | -.748 | 77.894 | .457 | -0.109 | 0.146 | -0.400 | 0.182 |

**2, Differences in post-test scores of English reading motivation between the experimental class and the control class**

| **Group Statistics** | | | | | |
| --- | --- | --- | --- | --- | --- |
| group | | N | Mean | Std. Deviation | Std. Error Mean |
| attention_posttest | experimental class | 40 | 3.58 | 0.92 | 0.15 |
|  | control class | 40 | 3.12 | 0.92 | 0.15 |
| relevance_posttest | experimental class | 40 | 3.42 | 0.91 | 0.14 |
|  | control class | 40 | 2.86 | 0.92 | 0.15 |
| confidence_posttest | experimental class | 40 | 3.93 | 0.73 | 0.12 |
|  | control class | 40 | 3.09 | 0.95 | 0.15 |
| satisfaction_posttest | experimental class | 40 | 3.65 | 0.94 | 0.15 |
|  | control class | 40 | 3.10 | 0.94 | 0.15 |
| totalmotivation_posttest | experimental class | 40 | 3.65 | 0.69 | 0.11 |
|  | control class | 40 | 3.04 | 0.75 | 0.12 |

| **Independent Samples Test** | | | | | | | | | | |
| --- | --- | --- | --- | --- | --- | --- | --- | --- | --- | --- |
|  | | Levene's Test for Equality of Variances | | t-test for Equality of Means | | | | | | |
|  |  | F | Sig. | t | df | Sig. (2-tailed) | Mean Difference | Std. Error Difference | 95% Confidence Interval of the Difference | |
|  |  |  |  |  |  |  |  |  | Lower | Upper |
| attention_posttest | Equal variances assumed | .037 | .848 | 2.271 | 78 | .026 | 0.47 | 0.21 | 0.06 | 0.88 |
|  | Equal variances not assumed |  |  | 2.271 | 77.999 | .026 | 0.47 | 0.21 | 0.06 | 0.88 |
| relevance_posttest | Equal variances assumed | .098 | .755 | 2.752 | 78 | .007 | 0.56 | 0.20 | 0.16 | 0.97 |
|  | Equal variances not assumed |  |  | 2.752 | 77.979 | .007 | 0.56 | 0.20 | 0.16 | 0.97 |
| confidence_posttest | Equal variances assumed | 2.315 | .132 | 4.447 | 78 | .000 | 0.84 | 0.19 | 0.47 | 1.22 |
|  | Equal variances not assumed |  |  | 4.447 | 73.321 | .000 | 0.84 | 0.19 | 0.47 | 1.22 |
| satisfaction_posttest | Equal variances assumed | .224 | .637 | 2.621 | 78 | .011 | 0.55 | 0.21 | 0.13 | 0.97 |
|  | Equal variances not assumed |  |  | 2.621 | 77.999 | .011 | 0.55 | 0.21 | 0.13 | 0.97 |
| totalmotivation_posttest | Equal variances assumed | .166 | .685 | 3.750 | 78 | .000 | 0.61 | 0.16 | 0.28 | 0.93 |
|  | Equal variances not assumed |  |  | 3.750 | 77.537 | .000 | 0.61 | 0.16 | 0.28 | 0.93 |

**3, Differences in English reading motivation between the pre-test and the post-test for the control class**

| Paired Samples Statistics | | | | | |
| --- | --- | --- | --- | --- | --- |
|  | | Mean | N | Std. Deviation | Std. Error Mean |
| Pair 1 | Pretest­_attention | 3.05 | 40 | 0.79 | 0.12 |
|  | attention_posttest | 3.12 | 40 | 0.92 | 0.15 |
| Pair 2 | Pretest_relevance | 2.78 | 40 | 0.77 | 0.12 |
|  | relevance_posttest | 2.86 | 40 | 0.92 | 0.15 |
| Pair 3 | Pretest_confidence | 2.96 | 40 | 0.95 | 0.15 |
|  | confidence_posttest | 3.09 | 40 | 0.95 | 0.15 |
| Pair 4 | pretestsatisfaction | 2.99 | 40 | 0.99 | 0.16 |
|  | satisfaction_posttest | 3.10 | 40 | 0.94 | 0.15 |
| Pair 5 | Pretest_total motivation | 2.94 | 40 | 0.64 | 0.10 |
|  | Total motivation_posttest | 3.04 | 40 | 0.75 | 0.12 |

| **Paired Samples Correlations** | | | | |
| --- | --- | --- | --- | --- |
|  | | N | Correlation | Sig. |
| Pair 1 | Pretest_attention & attention_posttest | 40 | .429 | .006 |
| Pair 2 | Pretest_relevance & relevance_posttest | 40 | .531 | .000 |
| Pair 3 | Pretest_confidence & confidence_posttest | 40 | .759 | .000 |
| Pair 4 | Pretest_satisfaction & satisfaction_posttest | 40 | .290 | .070 |
| Pair 5 | Pretest_total motivation & total motivation_posttest | 40 | .878 | .000 |

| **Paired Samples Test** | | | | | | | | | |
| --- | --- | --- | --- | --- | --- | --- | --- | --- | --- |
|  | | Paired Differences | | | | | t | df | Sig. (2-tailed) |
|  |  | Mean | Std. Deviation | Std. Error Mean | 95% Confidence Interval of the Difference | |  |  |  |
|  |  |  |  |  | Lower | Upper |  |  |  |
| Pair 1 | Pretest_attention - attention_posttest | -.06667 | .91894 | .14530 | -.36056 | .22722 | -.459 | 39 | .649 |
| Pair 2 | Pretest_relevance - relevance_posttest | -.08125 | .83087 | .13137 | -.34698 | .18448 | -.618 | 39 | .540 |
| Pair 3 | Pretest_confidence - confidence_posttest | -.12857 | .66046 | .10443 | -.33980 | .08265 | -1.231 | 39 | .226 |
| Pair 4 | Pretest_satisfaction - satisfaction_posttest | -.10833 | 1.14824 | .18155 | -.47556 | .25889 | -.597 | 39 | .554 |
| Pair 5 | Pretest_total motivation – total motivation_posttest | -.09621 | .35916 | .05679 | -.21107 | .01866 | -1.694 | 39 | .098 |

**4, Differences in English reading motivation between the pre-test and the post-test for the experimental class**

| **Paired Samples Statistics** | | | | | |
| --- | --- | --- | --- | --- | --- |
|  | | Mean | N | Std. Deviation | Std. Error Mean |
| Pair 1 | Pretest_attention | 2.93 | 40 | 0.72 | 0.11 |
|  | attention_posttest | 3.58 | 40 | 0.92 | 0.15 |
| Pair 2 | Pretest_relevance | 2.64 | 40 | 0.99 | 0.16 |
|  | relevance_posttest | 3.42 | 40 | 0.91 | 0.14 |
| Pair 3 | Pretest_confidence | 2.91 | 40 | 1.01 | 0.16 |
|  | confidence_posttest | 3.93 | 40 | 0.73 | 0.12 |
| Pair 4 | Pretest_satisfaction | 2.86 | 40 | 0.91 | 0.14 |
|  | satisfaction_posttest | 3.65 | 40 | 0.94 | 0.15 |
| Pair 5 | Pretest_total motivation | 2.83 | 40 | 0.67 | 0.11 |
|  | Total motivation_posttest | 3.65 | 40 | 0.69 | 0.11 |

| Paired Samples Correlations | |  |  |  |
| --- | --- | --- | --- | --- |
|  |  | N | Correlation | Sig. |
| Pair 1 | Pretest_attention & attention_posttest | 40 | 0.317 | 0.046 |
| Pair 2 | Pretest_relevance & relevance_posttest | 40 | 0.488 | 0.001 |
| Pair 3 | Pretest_confidence & confidence_posttest | 40 | 0.434 | 0.005 |
| Pair 4 | Pretest_satisfaction & satisfaction_posttest | 40 | 0.352 | 0.026 |
| Pair 5 | Pretest_total motivation & total otivation_posttest | 40 | 0.582 | 0.000 |

| **Paired Samples Test** | | | | | | | | | |
| --- | --- | --- | --- | --- | --- | --- | --- | --- | --- |
|  | | Paired Differences | | | | | t | df | Sig. (2-tailed) |
|  |  | Mean | Std. Deviation | Std. Error Mean | 95% Confidence Interval of the Difference | |  |  |  |
|  |  |  |  |  | Lower | Upper |  |  |  |
| Pair 1 | Pretest_attention - attention_posttest | -.65000 | .97241 | .15375 | -.96099 | -.33901 | -4.228 | 39 | .000 |
| Pair 2 | Pretest_relevance -relevance_posttest | -.78125 | .96108 | .15196 | -1.08862 | -.47388 | -5.141 | 39 | .000 |
| Pair 3 | Pretest_confidence - confidence_posttest | -1.02143 | .95943 | .15170 | -1.32827 | -.71459 | -6.733 | 39 | .000 |
| Pair 4 | Pretest_satisfaction - satisfaction_posttest | -.79167 | 1.05054 | .16610 | -1.12765 | -.45569 | -4.766 | 39 | .000 |
| Pair 5 | Pretest_total motivation – total motivation_posttest | -.81109 | .62175 | .09831 | -1.00993 | -.61224 | -8.251 | 39 | .000 |
