# Supplementary material for: Enhancing English reading motivation and performance via the ARCS model: an empirical study using the ARCS motivation scale
Source: Front Psychol. 2025 Oct 28;16:1499957. doi: 10.3389/fpsyg.2025.1499957 (PMC12602433; doi:10.3389/fpsyg.2025.1499957)
Supplement: Supplementary file 13 [file Table_8.doc]

**1, Differences in pre-test scores of English reading between the experimental and the control class**

| **Group Statistics** | | | | | |
| --- | --- | --- | --- | --- | --- |
| group | | N | Mean | Std. Deviation | Std. Error Mean |
| Reading_pretest | experimental class | 40 | 20.78 | 2.66 | .421 |
|  | control class | 40 | 20.88 | 3.16 | .500 |

| **Independent Samples Test** | | | | | | | | | | |
| --- | --- | --- | --- | --- | --- | --- | --- | --- | --- | --- |
|  | | Levene's Test for Equality of Variances | | t-test for Equality of Means | | | | | | |
|  |  | F | Sig. | t | df | Sig. (2-tailed) | Mean Difference | Std. Error Difference | 95% Confidence Interval of the Difference | |
|  |  |  |  |  |  |  |  |  | Lower | Upper |
| Reading_pretest | Equal variances assumed | 1.299 | .258 | -.153 | 78 | .879 | -.100 | .654 | -1.402 | 1.202 |
|  | Equal variances not assumed |  |  | -.153 | 75.811 | .879 | -.100 | .654 | -1.403 | 1.203 |

**2, Differences in post-test scores of English reading between the experimental and the control class**

| **Group Statistics** | | | | | |
| --- | --- | --- | --- | --- | --- |
| group | | N | Mean | Std. Deviation | Std. Error Mean |
| reading_posttest | experimental class | 40 | 23.15 | 2.90 | .458 |
|  | control class | 40 | 21.15 | 3.03 | .478 |

| **Independent Samples Test** | | | | | | | | | | |
| --- | --- | --- | --- | --- | --- | --- | --- | --- | --- | --- |
|  | | Levene's Test for Equality of Variances | | t-test for Equality of Means | | | | | | |
|  |  | F | Sig. | t | df | Sig. (2-tailed) | Mean Difference | Std. Error Difference | 95% Confidence Interval of the Difference | |
|  |  |  |  |  |  |  |  |  | Lower | Upper |
| reading_posttest | Equal variances assumed | .015 | .904 | 3.020 | 78 | .003 | 2.000 | .662 | .682 | 3.318 |
|  | Equal variances not assumed |  |  | 3.020 | 77.850 | .003 | 2.000 | .662 | .681 | 3.319 |

**3, Differences in the scores of English reading proficiency between the pre-test and the post-test for the control class**

|  | **Paired Samples Statistics** | | | | | |
| --- | --- | --- | --- | --- | --- | --- |
|  |  | | Mean | N | Std. Deviation | Std. Error Mean |
|  | Pair 1 | Reading_pretest | 20.88 | 40 | 3.16 | .500 |
|  |  | reading_posttest | 21.15 | 40 | 3.03 | .478 |

|  | | Paired Differences | | | | | t | df | Sig. (2-tailed) |
| --- | --- | --- | --- | --- | --- | --- | --- | --- | --- |
|  |  | Mean | Std. Deviation | Std. Error Mean | 95% Confidence Interval of the Difference | |  |  |  |
|  |  |  |  |  | Lower | Upper |  |  |  |
| Pair 1 | Reading_pretest - reading_posttest | -.275 | 3.274 | .518 | -1.322 | .772 | -.531 | 39 | .598 |
|  |  |  |  |  |  |  |  |  |  |

**4, Differences in** the scores of English reading proficiency between the pre-test and the post-test for the experimental class

| **Paired Samples Statistics** | | | | | |
| --- | --- | --- | --- | --- | --- |
|  | | Mean | N | Std. Deviation | Std. Error Mean |
| Pair 1 | Reading_pretest | 20.78 | 40 | 2.66 | .421 |
|  | reading_posttest | 23.15 | 40 | 2.90 | .458 |

| **Paired Samples Correlations** | | | | |
| --- | --- | --- | --- | --- |
|  | | N | Correlation | Sig. |
| Pair 1 | Reading_pretest & reading_posttest | 40 | .383 | .015 |

| **Paired Samples Test** | | | | | | | | | |
| --- | --- | --- | --- | --- | --- | --- | --- | --- | --- |
|  | | Paired Differences | | | | | t | df | Sig. (2-tailed) |
|  |  | Mean | Std. Deviation | Std. Error Mean | 95% Confidence Interval of the Difference | |  |  |  |
|  |  |  |  |  | Lower | Upper |  |  |  |
| Pair 1 | readingpretest - reading_posttest | -2.375 | 3.094 | .489 | -3.365 | -1.385 | -4.855 | 39 | .000 |
