# Supplementary material for: Enhancing English reading motivation and performance via the ARCS model: an empirical study using the ARCS motivation scale
Source: Front Psychol. 2025 Oct 28;16:1499957. doi: 10.3389/fpsyg.2025.1499957 (PMC12602433; doi:10.3389/fpsyg.2025.1499957)

**Predictability of motivational factors in English reading proficiency (RQ3)**

| **Descriptive Statistics** | | | |
| --- | --- | --- | --- |
|  | Mean | Std. Deviation | N |
| reading_posttest | 22.15 | 3.110 | 80 |
| attention_posttest | 3.3500 | .94266 | 80 |
| relevance_posttest | 3.1375 | .95126 | 80 |
| confidence_posttest | 3.5071 | .94293 | 80 |
| satisfaction_posttest | 3.3750 | .97273 | 80 |

|  | **Correlations** | | | | | | |
| --- | --- | --- | --- | --- | --- | --- | --- |
|  | | | reading_  posttest | attention_  posttest | relevance_  posttest | confidence_  posttest | satisfaction_  posttest |
| Pearson Correlation | | reading_posttest | 1.000 | .398 | .458 | .512 | .574 |
|  |  | attention_posttest | .398 | 1.000 | .627 | .470 | .574 |
|  |  | relevance_posttest | .458 | .627 | 1.000 | .645 | .573 |
|  |  | confidence_posttest | .512 | .470 | .645 | 1.000 | .470 |
|  |  | satisfaction_posttest | .574 | .574 | .573 | .470 | 1.000 |
| Sig. (1-tailed) | | reading_posttest |  | .000 | .000 | .000 | .000 |
|  |  | attention_posttest | .000 |  | .000 | .000 | .000 |
|  |  | relevance_posttest | .000 | .000 |  | .000 | .000 |
|  |  | confidence_posttest | .000 | .000 | .000 |  | .000 |
|  |  | satisfaction_posttest | .000 | .000 | .000 | .000 |  |
| N | | reading_posttest | 80 | 80 | 80 | 80 | 80 |
|  |  | attention_posttest | 80 | 80 | 80 | 80 | 80 |
|  |  | relevance_posttest | 80 | 80 | 80 | 80 | 80 |
|  |  | confidence_posttest | 80 | 80 | 80 | 80 | 80 |
|  |  | satisfaction_posttest | 80 | 80 | 80 | 80 | 80 |

| **Variables Entered/Removed^a^** | | | |
| --- | --- | --- | --- |
| Model | Variables Entered | Variables Removed | Method |
| 1 | satisfaction_posttest, confidence_posttest, attention_posttest, relevance_posttest^b^ |  | Enter |
| a. Dependent Variable: reading_posttest | | | |
| b. All requested variables entered. | | | |

| **Model Summary^b^** | | | | |
| --- | --- | --- | --- | --- |
| Model | R | R Square | Adjusted R Square | Std. Error of the Estimate |
| 1 | .636^a^ | .405 | .373 | 2.462 |
| a. Predictors: (Constant), satisfaction_posttest, confidence_posttest, attention_posttest, relevance_posttest | | | | |
| b. Dependent Variable: reading_posttest | | | | |

| **ANOVA^a^** | | | | | | |
| --- | --- | --- | --- | --- | --- | --- |
| Model | | Sum of Squares | df | Mean Square | F | Sig. |
| 1 | Regression | 309.497 | 4 | 77.374 | 12.762 | .000^b^ |
|  | Residual | 454.703 | 75 | 6.063 |  |  |
|  | Total | 764.200 | 79 |  |  |  |
| a. Dependent Variable: reading_posttest | | | | | | |
| b. Predictors: (Constant), satisfaction_posttest, confidence_posttest, attention_posttest, relevance_posttest | | | | | | |

| **Coefficients^a^** | | | | | | | | |
| --- | --- | --- | --- | --- | --- | --- | --- | --- |
| Model | | Unstandardized Coefficients | | Standardized Coefficients | t | Sig. | Collinearity Statistics | |
|  |  | B | Std. Error | Beta |  |  | Tolerance | VIF |
| 1 | (Constant) | 13.909 | 1.249 |  | 11.137 | .000 |  |  |
|  | attention_posttest | .005 | .401 | .001 | .012 | .991 | .536 | 1.865 |
|  | relevance_posttest | .083 | .446 | .026 | .187 | .852 | .426 | 2.350 |
|  | confidence_posttest | .984 | .390 | .298 | 2.522 | .014 | .567 | 1.763 |
|  | satisfaction_posttest | 1.337 | .372 | .418 | 3.591 | .001 | .585 | 1.709 |
| a. Dependent Variable: reading_posttest | | | | | | | | |

| **Collinearity Diagnostics^a^** | | | | | | | | |
| --- | --- | --- | --- | --- | --- | --- | --- | --- |
| Model | | Eigenvalue | Condition Index | Variance Proportions | | | | |
|  |  |  |  | (Constant) | attention_  posttest | relevance_  posttest | confidence_  posttest | satisfaction_  posttest |
| 1 | 1 | 4.861 | 1.000 | .00 | .00 | .00 | .00 | .00 |
|  | 2 | .044 | 10.480 | .73 | .06 | .17 | .01 | .04 |
|  | 3 | .041 | 10.885 | .02 | .10 | .13 | .38 | .34 |
|  | 4 | .032 | 12.347 | .03 | .58 | .01 | .07 | .61 |
|  | 5 | .022 | 14.790 | .22 | .25 | .70 | .54 | .00 |
| a. Dependent Variable: reading_posttest | | | | | | | | |

| **Residuals Statistics^a^** | | | | | |
| --- | --- | --- | --- | --- | --- |
|  | Minimum | Maximum | Mean | Std. Deviation | N |
| Predicted Value | 17.84 | 25.95 | 22.15 | 1.979 | 80 |
| Residual | -7.263 | 5.142 | .000 | 2.399 | 80 |
| Std. Predicted Value | -2.179 | 1.922 | .000 | 1.000 | 80 |
| Std. Residual | -2.950 | 2.088 | .000 | .974 | 80 |
| a. Dependent Variable: reading_posttest | | | | | |


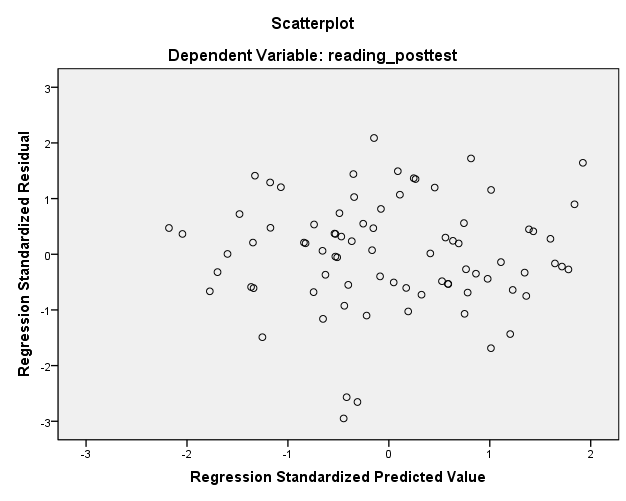

Supplement: Supplementary file 14 [file Table_9.doc]
